# Mechanistically linked serum miRNAs distinguish between drug induced and fatty liver disease of different grades

**DOI:** 10.1038/srep23709

**Published:** 2016-04-05

**Authors:** Zhichao Liu, Yuping Wang, Jürgen Borlak, Weida Tong

**Affiliations:** 1 Division of Bioinformatics and Biostatistics, National Center for Toxicological Research, U.S. Food and Drug Administration, Jefferson, Arkansas, USA; 2 Centre for Pharmacology and Toxicology, Hannover Medical School, Hannover, Germany

## Abstract

Hepatic steatosis is characterised by excessive triglyceride accumulation in the form of lipid droplets (LD); however, mechanisms differ in drug induced (DIS) and/or non-alcoholic fatty liver disease (NAFLD). Here we hypothesized distinct molecular circuits of microRNA/LD-associated target genes and searched for mechanistically linked serum and tissue biomarkers that would distinguish between DIS and human NAFLD of different grades. We analysed >800 rat hepatic whole genome data for 17 steatotic drugs and identified 157 distinct miRNAs targeting 77 DIS regulated genes. Subsequently, genomic data of N = 105 cases of human NAFLD and N = 32 healthy controls were compared to serum miRNA profiles of N = 167 NAFLD patients. This revealed N = 195 tissue-specific miRNAs being mechanistically linked to LD-coding genes and 24 and 9 miRNAs were commonly regulated in serum and tissue of advanced and mild NAFLD, respectively. The NASH serum regulated miRNAs informed on hepatic inflammation, adipocytokine and insulin signalling, ER-and caveolae associated activities and altered glycerolipid metabolism. Conversely, serum miRNAs associated with blunt steatosis specifically highlighted activity of FOXO1&HNF4α on CPT2, the lipid droplet and ER-lipid-raft associated PLIN3 and Erlin1. Altogether, serum miRNAs informed on the molecular pathophysiology of NAFLD and permitted differentiation between DIS and NAFLD of different grades.

The term hepatic steatosis refers to an intracellular accumulation of lipid droplets and is associated with an enlargement of the liver. Apart from drug induced steatosis over-nutrition is the most common cause of non-alcoholic fatty liver disease (NAFLD); it comprises diverse alterations in liver architecture and organ function and ranges from simple steatosis to non-alcoholic steatohepatitis (NASH), i.e. a condition accompanied by inflammation. If unresolved disease progression involves fibrosis, cirrhosis with risk for liver cancer^1^; however, mechanisms of disease progression are poorly understood. There is unmet need for non-invasive diagnostic markers that will permit disease monitoring and differentiation among different causes and grades of NAFLD.

Notably, based on current diagnostics, i.e. serum biochemistry, ultrasound or liver biopsies and by considering various patient populations and their associated co-morbidities a median prevalence for NAFLD of about 20% for the US and Europe has been reported. The incidence of NAFLD has substantially increased in recent years (http://www.bornnaturopathic.com/blog/health-articles/nonalcoholic-fatty-liver-disease-nafld-need-responsive/) and its progression to NASH is considered to be a “silent” killer^2^ with prognostic studies suggesting increased mortality in patients with NAFLD as compared to the general population^3^. Moreover, a strong link between NAFLD and cardiovascular disease has been established^4^. While the definitive diagnosis of NAFLD is based on histologic examination of liver biopsy inherent risks associated with the procedure limits its widespread use as a screening test (http://www.medscape.com/viewarticle/497001_5). Therefore, ultrasound and other imaging modalities have been encouraged for the diagnosis of NAFLD but apart from cost the procedures are cumbersome and cannot be used reliably for the grading of disease stages and differentiation between various causes of NAFLD including DIS.

Remarkably, the frequency of DIS is unknown and estimates of drug induced hepatic injury range considerably amongst different studies and countries with a crude incidence rate of 19.1/100,000 as was reported for the general population of Iceland^5^. Given that NAFLD has become an epidemic it would be highly desirable to have sensitive and specific serum markers for the rapid and cost effective screening of individuals at risk for NAFLD and/or DIS and to determine disease progression by minimal invasive or non-invasive means. Accordingly, therapeutic intervention strategies can be developed to prevent disease progression by actively screening patients with fatty liver disease. Although several risk factors for NAFLD have been identified and typically include type-2 diabetes & impaired glucose tolerance, clinical obesity, dyslipidaemia and eventually the metabolic syndrome, prolonged exposure of drugs can also be associated with DIS and NASH as reported for amidarone and other drugs^6^.

Importantly, drug induced hepatic steatosis is generally associated with more than one mechanism^7^ and either involves micro- or macrovesicular steatosis (e.g. valproic acid) and can be associated with ballooning degeneration of hepatocytes. Particularly at prolonged exposure to drugs cellular stress, as a result of impaired detoxification, and mitochondrial function becomes compromised thereby aggravating the condition. The main mechanism by which drugs cause steatosis were recently summarized and involve (a) inhibition of mitochondrial fatty acid beta oxidation (b) sequestration of CoA and/or L-Carnitine (c) inhibition of the mitochondrial respiration and modulation of the mitochondrial membrane potential (d) impairment of mitochondrial DNA replication (e) impaired peroxisome proliferator activated receptor (PPARα) transcriptional activity and other alterations in lipid homeostasis pathways^8^.

Due to the growing knowledge on the mechanisms of lipid droplet formation in hepatocytes and the possibilities to study mechanistically linked biomarkers we were particularly interested in studying molecular circuits and transcriptional responses of microRNA target genes in rat liver induced by steatotic drugs. The importance of non-coding RNAs (ncRNAs) in regulating lipid metabolism has just been unraveled with microRNAs (miRNAs) constituting a large family of small, approximately 21-nucleotide-long, non-coding RNAs that have emerged as key post-transcriptional regulators of gene expression^9^. By base pairing to mRNAs, miRNAs mediate translational repression or mRNA degradation^10^. In mammals, miRNAs are predicted to control the activity of approximately 30% of all protein-coding genes, and have been shown to participate in the regulation of almost every cellular process investigated so far^10^. Recent research highlighted the critical functions of miRNAs in regulating diverse biological processes including the immune system, cell proliferation, differentiation and development, cancer and cell cycle^11^,^12^,^13^ and several miRNAs function in a tissue-specific manner^14^,^15^ as determined for NAFLD^16^.

Altogether, the primary objective of our study was to define mechanistically linked serum miRNAs that would distinguish between DIS and NAFLD of different grades. We hypothesized distinct transcriptional regulations of co-expressed miRNA/target genes in rat liver induced by steatotic drugs and utilized our knowledge of about 200 genes being mechanistically involved in various pathophysiological processes during fatty liver disease as recently reported^17^. By interrogating the publically available Open TG-GATEs database the dose and time dependent effects of 17 DIS causing drugs could be investigated and by studying whole genome transcript expression data of human NAFLD co-expressed miRNA-target genes in DIS and NAFLD were ascertained. Importantly, the identification of mechanistically linked miRNA serum biomarkers for DIS and NAFLD of different grades provide opportunities for its early detection and the monitoring of disease progression and will aid drug selection in preclinical development programs.

## Methods and Materials

### Chemicals

Table 1 informs on drugs and chemicals used to induce hepatic steatosis.

### Ethical statement and animal experimentation

The animal studies conformed to the Guide for the Care and Use of Laboratory Animals (The National Academy Press, Washington, D.C., 1996) and were conducted by the Japanese National Institute of Health Science, the National Institute Biomedical Innovation and pharmaceutical companies after obtaining approval from the Ethics Review Committee for Animal Experimentation of the National Institute of Health Sciences, Japan^18^. Specifically, male Crj:CD Sprague Dawly (SD) rats at the age of 6 weeks were given either a single low, middle and high dose and were sacrificed at 3, 6, 9 and 24 hours or received repeated treatments for 3, 7, 14 and 28 days. At the end of the study the liver was removed and next to histopathology representative samples were stored at −80 °C for further analysis. Further information regarding administration routes and dosage details are available on the TG-GATE database (http://toxico.nibio.go.jp). In this study, only *in vivo* data were used, which included both single and repeated dosing experiments.

### Histopathology

Formalin fixed paraffin-embedded tissue blocks were sectioned to approximately 3 μm thickness and stained with H and E. Hepatic steatosis was confirmed by board certified pathologists after evaluating randomly selected fields at 20X magnification.

### Data processing

A total of 808 Affymetrix GeneChip Rat Genome 230 2.0 raw data (CEL files) of 17 steatotic drugs and matched controls were retrieved from TG-GATE (http://toxico.nibio.go.jp/english/index.html). The data were analyzed using the robust multi-array average (RMA) methodology in the Bioconductor, R package for background-adjusted, normalized, and log-transformed perfect matched values of individual probes from the Array. (http://www.bioconductor.org/packages/release/bioc/html/BufferedMatrixMethods.html).

The fold change for each compound was calculated by comparing treatment data and matched controls; the student t test with multiple testing corrections was employed to calculate *p* values for each gene. Differential expressed genes (DEGs) were considered for further analysis by using the criterion fold change more than 1.5 and Benjamini & Hochberg adjusted *p* value less than 0.05.

### Genes involved in lipid droplet formation of hepatocytes

Based on our previous works we considered approximately 200 steatosis related genes^17^ which code for lipid transport & lipogenesis, lipid droplet associated proteins such as the perilipins, glucose & fatty acid metabolism and signalling events. Details are given in supplementary Table S1 and the gene symbols were mapped to Entrez Gene ID based on NCBI gene annotation (http://www.ncbi.nlm.nih.gov/gene/).

### MiRNA-gene target predictions

MiRNA targets were predicted using the miRTarBase repository http://mirtar.mbc.nctu.edu.tw/human/index.php)^19^. The database defines experimentally proven miRNA-gene target relationships. DEGs for 17 steatotic drugs (see above) and data from N = 4 independent publically available genomic data sets of N = 105 cases of human liver NAFLD and N = 32 controls (see below “translational research”) were mapped to miRTarBase. A summary of the miRNA-gene relationships is given in supplementary Table S2 and the data were compared to N = 207 cases of experimentally verified serum miRNA profiles. Furthermore, consensus prediction of miRNA targets involved 12 different computational algorithms, i.e. DIANA-microTv4.0 [ http://diana.imis.athena-innovation.gr/DianaTools/index.php?r =  microtv4/index], DIANA-microT-CDS [ http://diana.imis.athena-innovation.gr/DianaTools/index.php?r =  microT_CDS/index], miRanda [ http://www.microrna.org/microrna/home.do], mirBridge [PMID:20385095], miRDB [ http://mirdb.org/miRDB], miRmap [ http://mirmap.ezlab.org], miRNAMap [ http://mirnamap.mbc.nctu.edu.tw], PicTar2 [ https://www.mdc-berlin.de/10440258/en/research/research_teams/systems_biology_of_gene_regulatory_elements/projects/pictar], PITA [ http://genie.weizmann.ac.il/pubs/mir07/mir07_data.html], RNA22 [ https://cm.jefferson.edu/rna22/Interactive/], RNAhybrid [ http://bibiserv.techfak.uni-bielefeld.de/rnahybrid] and TargetScan [ http://www.targetscan.org/].

Bona fide miRNA targets were defined when predicted by at least nine different algorithms.

### KEGG pathway analysis

The Database for Annotation, Visualization, and Integrated Discovery (DAVID) (http://david.abcc.ncifcrf.gov/) was used to identify Kyoto Encyclopedia of Genes and Genomes (KEGG) annotated pathways. The Benjamini-Hochberg (BH) adjusted *p* value of ≤0.05 was applied as a cut-off to determine significantly enriched pathways.

### Protein–protein interaction

The STRING version 9.1 database was used to determine protein-protein interactions amongst steatosis regulated DEGs^20^. Only interactions with confidence scores >0.4 were considered.

### Network analysis

Network construction of co-regulated miRNA-target genes induced by drugs was based on the MCODE method^21^. The parameters in MCODE were set as default with NodeScoreThreshold = 0.2, *K*-coreThreshold = 2 and MaxDepth = 100.

### Chemical structure similarity

The structural similarity among steatosis inducing drugs/chemicals was determined by using functional class fingerprints (FCFPs) with a radius of FCFP_4 as chemical descriptors to calculate Tanimoto coefficients. The data were subsequently analyzed using the software Pipeline Pilot 8.0 (Accelrys, http://accelrys.com/).

### Translational research

**Figure 1:**
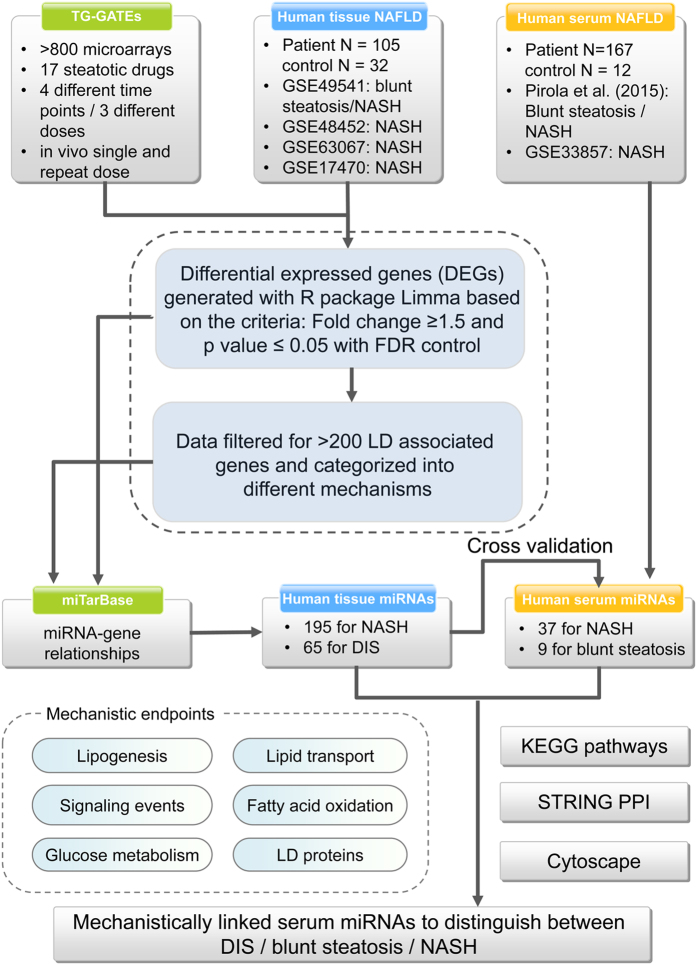
Flowchart of experimental strategies and associated data analysis.

The genomics of human NAFLD involved whole genome data sets GSE48452, GSE63067, and GSE17470 that were retrieved from the GEO database (http://www.ncbi.nlm.nih.gov/geo/). Alike, NAFLD regulated serum miRNAs were studied by analyzing the data set GSE33857 and the results reported by Pirola *et al*.^22^ (Fig. 1 and Table 2). Furthermore, gene expression data from N = 72 NAFLD patients, i.e. N = 40 with mild NAFLD, fibrosis stage 0–1 and N = 32 with advanced NAFLD, fibrosis stage 3–4 were retrieved from the GEO database (GSE49541). Importantly, the study of Moylan *et al*.^23^ (GEO data set GSE49541) did not involve healthy control cases^23^. Therefore, DEGs were calculated by considering whole genome hepatic gene expression data of N = 7 healthy controls regained from the López-Vicario *et al*. study^24^. In their study (GEO data set GSE37031) NASH patients and healthy controls were analyzed with the same microarray platform as utilized in the study of Moylan *et al*.^23^. In order to eliminate a batch effect, the CEL files from the two studies were processed together using the robust multi-array average (RMA) methodology and DEGs for mild NAFLD and advanced NAFLD were generated with the R limma package. Altogether, genomic data sets of N = 105 cases of human liver NAFLD and N = 32 controls were considered and DEGs were generated with the GEO2R (http://www.ncbi.nlm.nih.gov/geo/geo2r/) software with the filtering criteria fold change >1.5 and Benjamini & Hochberg adjusted *p* value < 0.05.

Tissue regulated miRNA were predicted from whole genome hepatic gene expression changes by querying the miRTarBase repository (see above). Additionally, the whole genome DEG data were filtered for N = 200 genes mechanistically involved in lipid droplet formation of hepatocytes (supplementary Table S1 and [18]) and both approaches were subsequently mapped to miRTarBase to identify associated miRNAs. Moreover, prediction of gene targets was based on miRNA consensus among 9 out of 12 different algorithms (see above).

The tissue regulated miRNA were validated by considering experimentally verified serum miRNA profiles obtained from N = 12 healthy controls and N = 167 NAFLD patients samples. Note, for all patients liver biopsies were obtained to permit an assessment of grades of fatty liver disease and included N = 66 cases of benign steatosis and N = 94 NASH cases of different grades^22^. A further independent data set of serum microRNA expression in human NAFLD (GEO Accession: GSE33857) encompassing N = 12 controls with normal liver function and N = 7 NASH cases was considered^25^.

## Results

The study design and work flow is depicted in Fig. 1 and statistically significant differentially expressed genes (DEGs) were identified in liver tissue of rats after single and repeated treatment with 17 steatotic drugs for up to 28 days. Subsequently, the data were filtered for genes known to be mechanistically linked to lipid droplet formation in hepatocytes (see supplementary Table S1) and miRNAs targeting DIS regulated genes were obtained by interrogating the experimentally verified miRTar database. Thus, the data analysis focused on (1) an identification of miRNAs targeting LD associated genes in hepatic steatosis; (2) time/dose effects in drug induced steatosis (3) an identification of commonly regulated tissue and serum miRNAs for its utility as serum biomarker and (4) serum miRNAs to distinguish DIS and human NAFLD of different grades.

### Prediction of tissue regulated miRNA in drug induced steatosis

**Figure 2:**
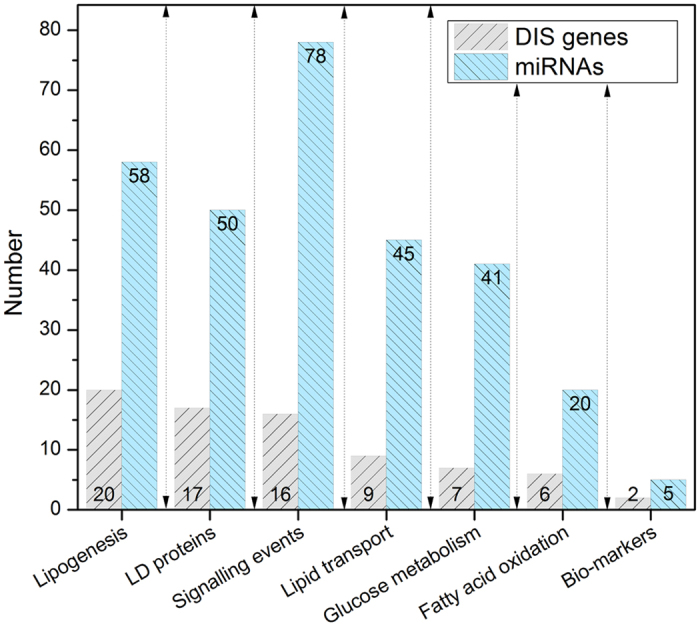
Co-regulation of miRNA and gene targets in drug induced steatosis. The histogram depicts the number of miRNAs involved in the regulation of N = 77 DIS gene targets. The data are grouped according to distinct processes in the pathophysiology of lipid droplet formation in hepatocytes. The DIS signature genes are given in supplementary Table S3 and are mechanistically linked to fatty liver disease.

Computational analysis of 17 steatotic drugs involved >800 whole genome microarrays and the subsequent filtering of DEGs for 200 hepatic steatosis related genes (supplementary Table S2). This revealed 409 miRNA-gene associations and consisted of 157 distinct miRNAs and 77 genes. The mapping of significant DEGs to different processes in lipid droplet formation (Fig. 2) discovered >20 genes to be regulated in the process of lipogenesis while KEGG pathways analysis highlighted significant enrichment for glycerol lipid and glycerophospholipid metabolism (Table 3) as exemplified by the significant regulation of DGAT1, i.e. a key enzyme catalyzing the terminal step in triacylglycerol synthesis as well as AGPAT isoforms involved in the metabolism of lysophosphatidic acid to phosphatidic acid (PA). The latter are important signalling molecules but also function as building block intermediates in lipid biosynthesis. Other highly regulated genes include fatty acid synthase as well as lecithin cholesterol acyltransferase, the desaturase FADS1, FADS2, the transcription factors PPARγ, SREBF1 and the SREBP cleavage protein SCAP, the glucosylceramide synthase and various annexins to influence Ca2+ dependent phospholipid binding to modify cytoskeletal dynamics.

**Figure 3:**
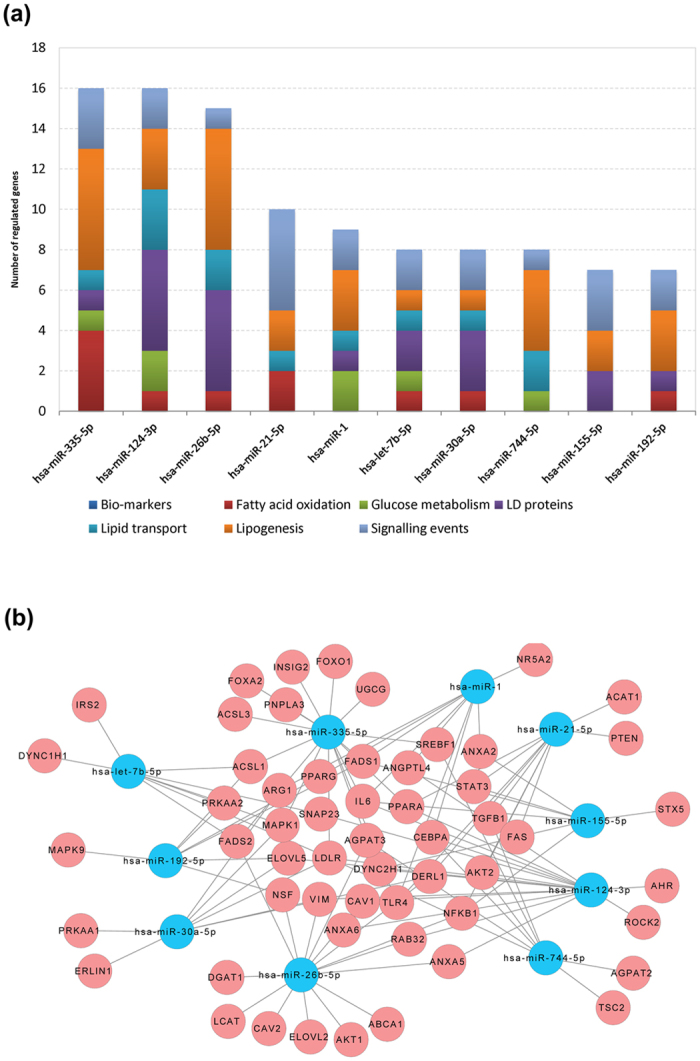
DIS associated gene regulations by miRNAs. **(a)** The histogram depicts the top 10 ranked miRNAs and their distribution amongst different lipid droplet associated processes; **(b)** Cytoscape network analysis of mechanistically linked LD-associated gene regulations of the top 10 ranking miRNAs. The blue and pink colored nodes refer to miRNAs and LD-associated genes, respectively.

The frequency by which individual miRNAs target steatosis related genes was considered; Fig. 3a depicts hsa-mir-335-5p and has-miR-124-3p as top ranking. These are involved in the regulation of genes coding for lipogenesis (n = 6/3), signalling events (n = 3/2), lipid transport (n = 1/3), lipid droplet associated proteins (n = 1/5), fatty acid oxidation (n = 4/1) and glucose metabolism (1/2). The network of LD-associated gene regulations among the top 10 ranking miRNAs is given in Fig. 3b and Table 4 summarizes independent studies to confirm the top ranking miRNAs as significantly regulated in animal and human NAFLD.

**Figure 4:**
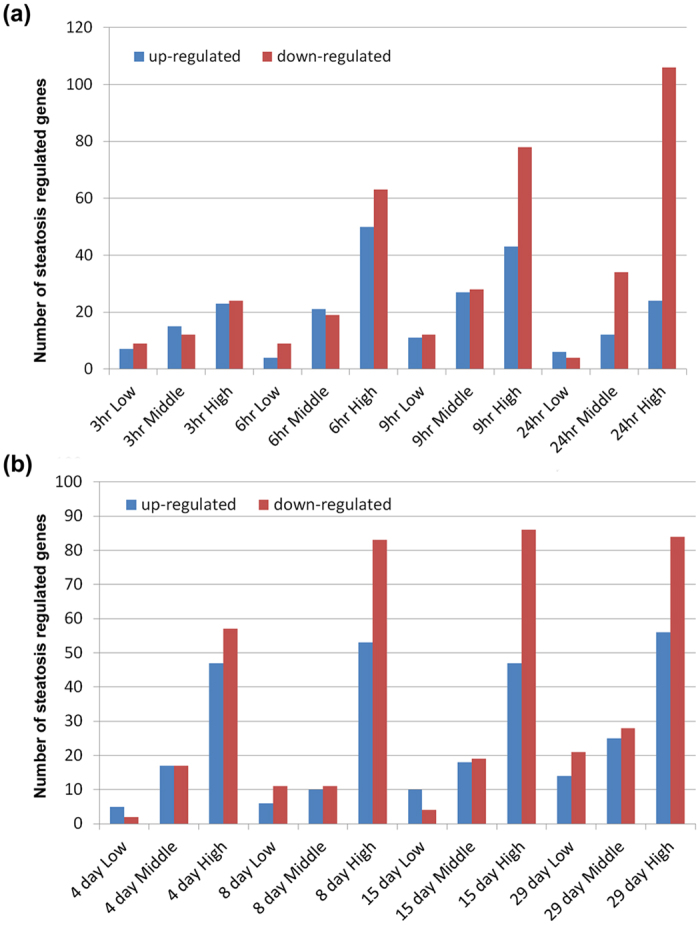
Cumulative DIS associated gene regulations after single and repeated treatment of rats with N = 17 steatotic drugs. **(a)** Single dose treatment. **(b)** Repeated dose treatment for up to 28 days. Details regarding the animal protocol are given in the Material and Method section and in^18^.

**Figure 5:**
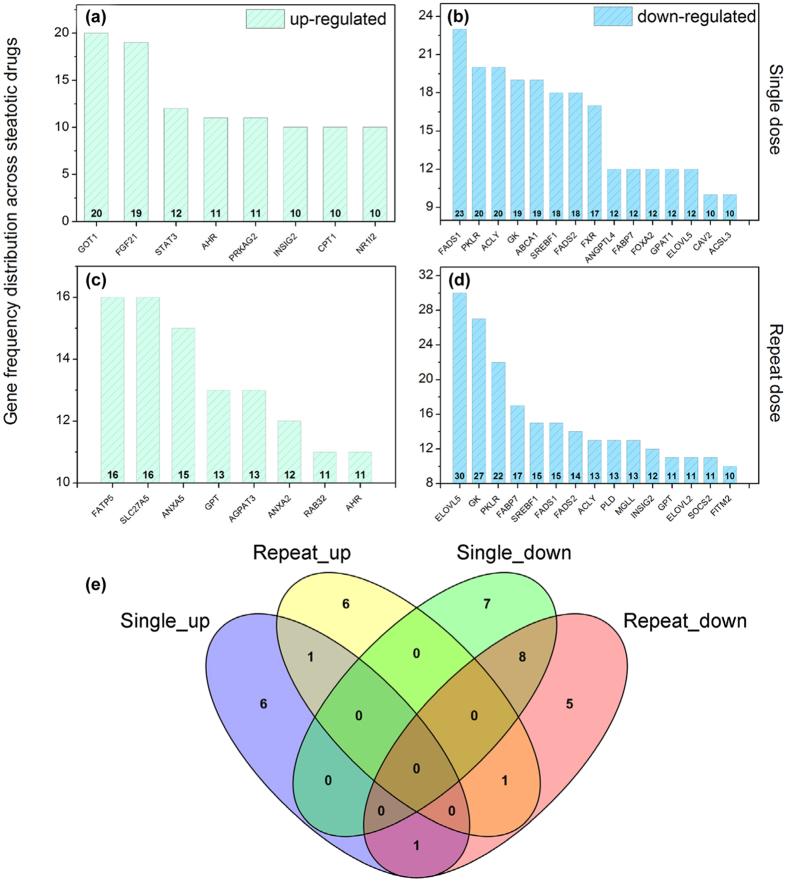
Frequency distribution of top 10 ranked DIS signature genes. Panels **(a,b)** depict the frequency distribution of up- or down regulated genes after single treatment of rats with N = 17 steatotic drugs; Panels **(c,d)** describe the frequency distribution of up- or down regulated genes after repeated treatment of rats with N = 17 steatotic drugs for 28 days; **(e)** Venn diagram of common regulated genes among the different treatment conditions (see panels **a**–**d)**. Details regarding the animal protocol are given in the Material and Method section and in^18^.

Moreover, the drug induced dose and time dependent regulations were considered and as shown in Fig. 4 the higher dose majorly affected repression rather than induction of gene transcription after single and repeated drug treatment. In an effort to define commonly regulated genes (frequency >10) the different drug, dose and time constellations were considered (Fig. 5a–d) and compared by constructing a Venn diagram to obtain information on the overlap of genes among the various considerations (Fig. 5e). This defined the aryl hydrocarbon receptor (AhR) as commonly up-regulated after single and repeat dosing with 17 different drugs while the genes FADS1, PKLR, ACLY, GK, SREBF1, FADS2, FABP7, and ELOVL5 were commonly repressed in expression. Note, the molecular functions of the aryl hydrocarbon receptor and the liver X receptor in the regulation of metabolic homeostasis is the subject of intense research; evidence was obtained for the aryl hydrocarbon receptor to induce hepatic steatosis via the up-regulation of the fatty acid transporter CD36^18^,^26^. Additional miRNAs involved in the regulation of the AhR include has-mir-26a-5p, hsa-mir-130b-3p, has-mir-124-3p, has-miR-625-5p and has-miR-98-5p with proven experimental evidence for their participation in the regulation of genes coding for lipid transport most notable CD36, fatty acid binding proteins FABP1, FAB6, FAB7, low density lipoprotein receptor, RXRß and others based on miRTarBase data analysis and PubMed searches.

As depicted in Fig. 5e eight genes were commonly down regulated after single and repeated treatment of rats with 17 steatotic drugs and included FADS1, PKLR, ACLY, GK, SREBF1, FADS2, FABP7 and ELOVL5.

We also considered the chemical structure similarity amongst 17 steatotic drugs by calculating the Tanimoto coefficient and found it to be insignificant. Nonetheless, when the frequency of DIS associated miRNAs were considered hsa-miR-335 was most frequently regulated across a wide range of dissimilar drugs.

Lastly, when miRNAs obtained from consensus predictions were compared with results obtained from miRTarBase queries a total of 35 miRNAs were in common (supplementary Fig. S1).

### Molecular circuits of co-expressed miRNA-gene targets in DIS

Based on the built miRNA- steatosis and drug-steatosis gene interactions, we considered drug, dose and time associations (supplementary Table S3) and subsequently constructed protein-protein-interaction networks by probing the STRING PPI database version 9.1. As a result, 128 and 162 PPIs corresponding to 48 and 50 genes were obtained after single and repeated drug treatment of rats with 17 steatotic drugs (supplementary Table S4). Furthermore, by utilizing the MCODE algorithm the endoplasmic reticulum localized fatty acid desaturases FADS1, FADS2 and the fatty acid elongases ELOVL2 and ELOVL5 were identified as commonly regulated after single and repeat drug treatment. Consistent with previous findings expression of these genes is repressed and confer functional diversity through changes in chain length and degree of unsaturation in the process of lipogenesis.

**Figure 6:**
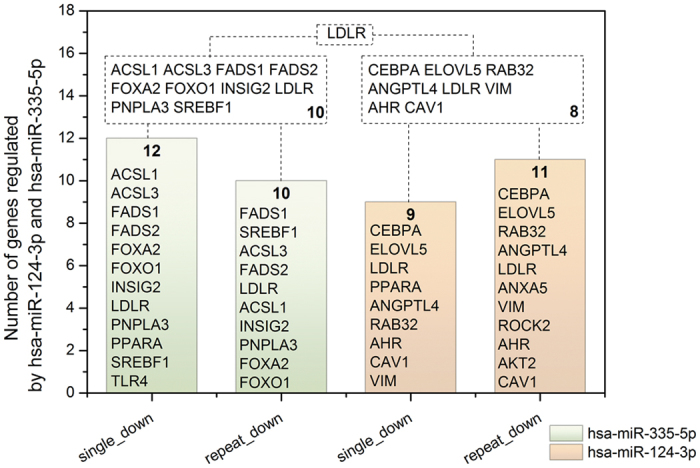
Transcriptional repression of lipid droplet associated genes by has-miR-3355p and has-miR-124-3p. Green and orange colored bars refer to transcriptional repression of genes induced by has-miR-335-5p and hsa-miR-124-3p after single and repeat treatment of rats with N = 17 steatotic drugs.

Alike, individual networks for top regulated miRNAs were considered and as an example Fig. 6 depicts hsa-miR-124-3p and hsa-miR-335 regulated DIS genes after single and repeated treatment of rats with 17 drugs for up to 28 days. Transcriptional regulation involved 12, 10, 9 and 11 repressed genes after single and repeated treatments, respectively and the LDL receptor was commonly targeted amongst these miRNAs irrespective of the treatment conditions. Note, up-regulation of liver tissue specific hsa-miR-335 was reported for murine models of NAFLD, e.g. the leptin deficient ob/ob, the leptin-receptor-deficient db/db and KKAy44 mice. It is of considerable importance that hsa-miR-335 is a major regulator for lipid accumulation and adipose tissue differentiation in mice^27^ and the transcription factors FOXO1 and FOXA2, the insulin induced gene 2 and the low density lipoprotein receptor LDLR were equally regulated by miR-335. For its frequent regulation by different drugs and its important role in lipid homeostasis has-miR-335 should be considered as a DIS biomarker in drug safety evaluation. Similarly, among the miR-124 target genes was the Rho-associated, coiled-coil containing protein kinase 2. Recent evidence suggests repression of ROCK proteins to prevent hepatic steatosis by reducing lipid synthesis while pharmacological inhibition of ROCK prevented severe ischemia/reperfusion injury in steatotic fatty liver allografts^28^. The observed repression of ROCK transcripts can therefore be considered as an adaptive response to drug induced steatosis.

### Translational research

**Figure 7:**
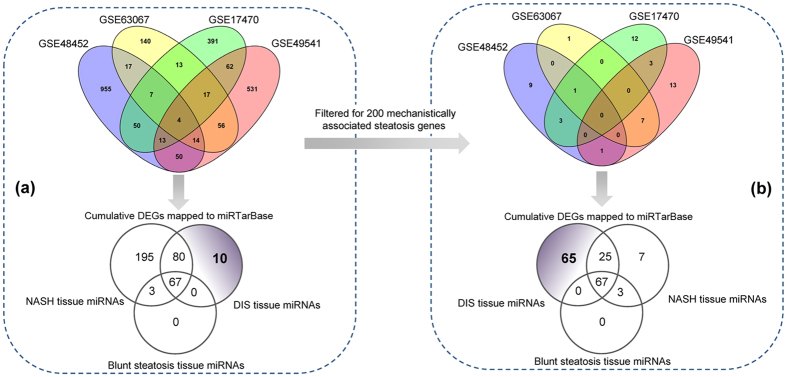
Venn diagram of differentially expressed genes in DIS and NAFLD. **(a)** The publically available human NAFLD gene expression data (GEO accession number: GSE48452, GSE63067, GSE17470 and GSE49541) were analysed as described in the Material and Method section. DEGs were mapped to the miRTar database and compared to DIS associated miRNAs after single and repeated treatment of rats with N = 17 steatotic drugs for up to 28 days. **(b)** DEGs obtained from the publically available data sets GSE48452, GSE63067, GSE17470 and GSE49541 were filtered for N = 200 mechanistically linked LD-associated gene regulations (see supplementary Table S1) and mapped to the miRTar database. The data were compared with DIS associated miRNA regulations.

As depicted in Fig. 1 N = 4 independent studies were retrieved from the GEO database. Next to determining differentially expressed genes (DEGs) the data were mapped to miRTarBase and the results were compared with predicted DIS regulated miRNAs for 17 drugs after single and repeated treatment of rats for up to 28 days. Among 157 DIS regulated miRNAs 147 were regulated in common with human NAFLD (Fig. 7a). Alike, when human NAFLD data were filtered for 200 genes mechanistically linked to hepatic steatosis (Fig. 7b) 92 DIS regulated miRNAs were commonly regulated; vice versa 10 (see Fig. 7a) or 65 (see Fig. 7b) miRNA were predicted to be DIS tissue specific. Altogether, a total of 195 or 7 NASH specific miRNAs were identified by either considering whole genome human liver tissue DEGs or by filtering DEGs for 200 mechanistically linked genes. This analysis also revealed the lack of blunt steatosis specific tissue regulated miRNAs.

### Serum biomarker candidates

**Figure 8:**
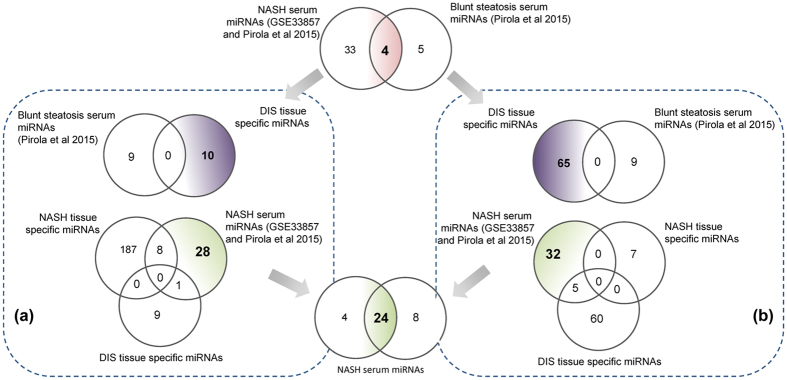
Translational biomarkers. **(a)** Upper panel: Venn diagram of blunt steatosis serum regulated miRNAs and DIS tissue regulated miRNAs. Note, the DIS tissue specific miRNAs are based on the data given in Fig. 7a. Lower panel: Comparison of NASH tissue and NASH serum regulated miRNAs with DIS tissue specific miRNAs. **(b)** Upper panel: Venn diagram of DIS tissue and blunt steatosis serum regulated miRNAs. Note, the DIS tissue specific miRNAs are based on the data given in Fig. 7b (i.e. the whole genome data was filtered for N = 200 mechanistically linked LD-associated gene regulations (see supplementary Table S1). Lower panel: Comparison of NASH and DIS tissue miRNAs with NASH serum regulated miRNAs.

Based on whole genome gene expression profiling and by considering commonalities amongst independent publically available studies NASH and DIS specific miRNAs were analyzed. Their regulation was compared to experimentally verified serum miRNA profiling studies and as shown in Fig. 8 a total of 37 and 9 miRNAs were significantly changed in serum miRNA profiling studies of NASH and cases of blunt steatosis, respectively (Fig. 8 upper panel). Subsequently, DIS tissue specific miRNAs were compared to blunt steatosis regulated serum miRNAs. This revealed none of the miRNA to be in common thus evidencing specificity of the performed analysis (Fig. 8a,b). Additionally, miRNAs commonly regulated in liver tissue amongst 4 independent NASH studies were compared with DIS tissue and serum regulated miRNAs. Such an analysis discovered 28 and 32 NASH serum specific miRNAs by either considering total DEGs (approach A) or DEGs filtered for 200 mechanistically linked hepatic steatosis genes (approach B). The Venn diagram shown in the lower panel of Fig. 8 informs on 24 common miRNAs to demonstrate that the hypothesis driven approach (see panel b) yielded 65% of experimentally verified serum regulated miRNAs in NAFLD.

**Figure 9:**
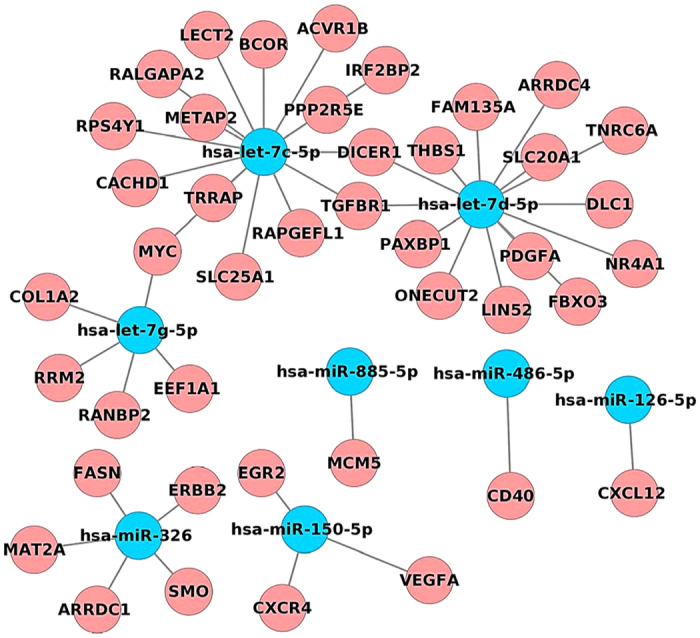
Commonly regulated tissue and serum miRNAs in NASH. Eight miRNAs were identified as commonly regulated when independent NASH tissue and serum profiling studies are compared.

**Figure 10:**
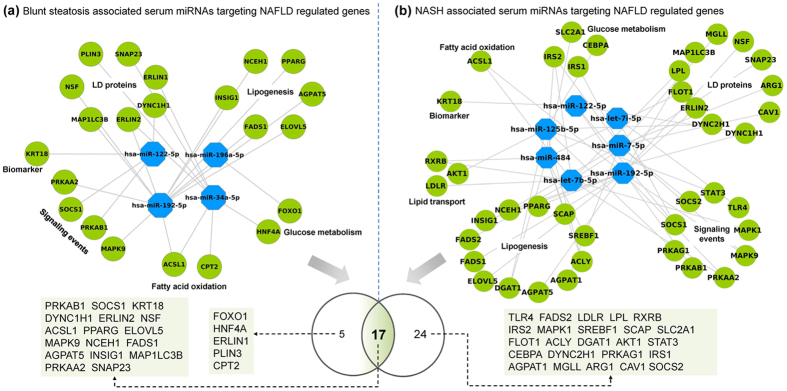
Serum regulated miRNAs distinguish between blunt steatosis and NASH. Significantly regulated serum miRNAs inform on pathophysiological processes in fatty liver disease and permit differentiation between NAFLD of different grades.

Our study highlights 8 common miRNAs when independent NASH tissue and serum profiling studies are compared and includes the miRNAs: hsa-let-7c-5p, hsa-let-7d-5p, hsa-let-7g-5p, hsa-miR-326, hsa-miR-150-5p, has-miR-885-5p, has-miR-486-5p and has-miR-126-5p. The network of regulated genes by these miRNAs is depicted in Fig. 9 and the results given in Fig. 10 evidence differentiation between NAFLD of different grades. Specifically, miRNAs regulated in blunt steatosis were analysed for target genes by mapping them to miRTarBase; the resultant target genes were compared to DEGs determined in hepatic tissue of NAFLD patients and to the list of 200 genes mechanistically involved in fatty liver disease (supplementary Table S2). Accordingly, a network was constructed and visualised with Cytoscape and 4 miRNAs were found to influence transcriptional expression of 22 steatosis related genes. This included the lipid droplet associated protein PLIN3, the transcription factors FOXO1, HNF4a and PPARγ, the ER lipid raft associated ERLIN1 and 2, the lysophosphatidic acid acyltransferase, protein and mitogen activated kinases and SNAP23 involved in the lipid fusion process. Using the same approach NASH regulated serum miRNAs were analysed and a total of 7 miRNAs were identified to target 41 fatty liver disease regulated genes. Here, significant enrichment for adipocytokine (i.e. AKT1, IRS2, ACSL1, RXRB, PRKAG1, SLC2A1, PRKAB1, MAPK9, PRKAA2, IRS1, STAT3) and insulin signalling pathways (SREBF1, AKT1, MAPK1, IRS2, SOCS2, PRKAG1, SOCS1, FLOT1, PRKAB1, MAPK9, PRKAA2, IRS1), regulation of suppressor of cytokine (SOCS1 & SOCS2) and Toll-like receptor 4 signalling were important findings. Further notable findings were miRNAs involved in the regulation of the LDL receptor, various transcription factors (RXRB, STAT3), calveole and fusion associated proteins CAV1, SNAP23, Flotilin, the monoglyceride lipase 1 and the microtubule-activated ATPase dynein 1 which functions in vesicle transport. Additionally, a total of 17 genes were regulated in common when serum miRNAs regulated in blunt steatosis and/or NASH were compared (Fig. 10 lower panel). Taken collectively, circulating miRNAs provide opportunities for disease diagnostic that may be extended to drug therapeutic monitoring. Table 4 compiles independent experimental evidence for the top ten DIS regulated miRNAs in serum or tissue of animal and human NAFLD. For instance, the anti-fibrotic miR-335-5p as well as miR-30a-5p are known to repress lipid biosynthesis, lipoprotein secretion and autophagasome formation and were significantly regulated as was mir-21 and mir-335 which are mechanistically linked to hepatic triglyceride and cholesterol metabolism.

## Discussion

A recent white paper by the American Association for the Study of Liver Diseases, the American College of Gastroenterology and the American Gastroenterological Association provided guidance for the diagnosis and the management of NAFLD^29^. In this report the current limitations in the diagnosis of NAFLD are summarized with serum biochemistries frequently failing to be informative as they can be within normal range or are insufficiently sensitive to reflect grades of disease; alike liver ultrasound imaging requires skilled healthcare professionals and might be obscured by gross morphologic changes other than NAFLD. Furthermore, ultrasound imaging does not inform on some important diagnostic and prognostic factors in NAFLD. Therefore, the diagnosis and assessment of progressive fatty liver disease remains challenging; nonetheless the discoveries of molecular disease markers carry the hope to replace liver biopsies by novel blood and/or urinary tests.

The significant role of miRNAs in regulating lipid metabolism was the subject of several reviews^30^; however, only a few studies investigated the regulation of miRNAs in drug induced steatosis^31^. As of today it remains uncertain whether miRNAs known to regulate cholesterol and fatty acid metabolism and lipid droplet formation are the same in DIS and NAFLD.

In the present study whole genome transcript expression changes were examined to investigate tissue regulated miRNAs and to permit construction of miRNA-gene networks specific for fatty liver disease and DIS. Based on diverse computational approaches distinct circuits of co-expressed microRNA target genes in NAFLD and DIS were postulated and validated by comparing tissue with serum regulated miRNAs. This enabled an identification of mechanistically linked serum biomarker candidates for DIS and NAFLD of different grades. Table 5 compiles a summary of regulated miRNAs in human NAFLD (Table 5A) and in animal models of NAFLD (Table 5B).

The present study highlights the importance of mechanistically linked lipid droplet associated gene regulations to inform on the pathophysiological state of fatty liver disease with serum miRNA profiling representing a rapid and cost effective means for an identification of diagnostic biomarkers.

Importantly, fatty liver disease is a clinico-pathologic feature of drug induced liver injury (DILI) and is typically seen with drugs such as amiodarone, perhexiline, carbamazepine amongst others. It results from an imbalance between xenobiotic defense and adaptation leading to ER/mitochondrial stress, unfolded protein response and liver injury. Recently, whole genome transcript profiling of drug induced steatosis was reported and comprehensive information on mechanistically linked gene expression changes was obtained for a wide range of drugs causing either steatosis and/or phospholipidosis while an identification of signature genes encourage their use in preclinical screening assays^18^.

Unfortunately, commonly used markers of liver injury lack specificity and do not distinguish between DIS and NAFLD of different grades but rely primarily on serum biochemistries, for instance by assaying serum alanine and aspartate aminotransferase activities as a means to examine the metabolic competence of the liver. In recent years molecular biomarkers of hepatotoxicity and their potential to improve understanding and management of DILI have been intensely researched as summarized in a recent research highlight^32^,^33^.

To overcome current limitations mechanistically based biomarkers have been advocated and await translational research to better predict prognosis and outcome than currently used clinical biochemistry parameters^34^. Serum miRNAs may therefore be employed to predict individuals at risk for developing fatty liver disease and in our previous research we identified serum acute phase reactants to define healthy individuals at risk for acetaminophen induced liver injury prior to drug treatment^35^, while the study of Yamaura and co-workers^31^ identified miRNAs indicative of the pattern of liver injury that is steatosis versus NASH, cholestatic disease and acute versus chronic hepatocellular injury using a range of drugs and dietary animal models. Indeed, when the data of the Yamaura’s study were compared with findings of the present study several miRNAs were regulated in common and included for blunt steatosis miR-10b and miR-183; similarly with NASH the miRNAs miR-17, miR148b-5p and miR-197 were commonly regulated thus providing independent evidence for their diagnostic utility in animal studies.

In the present study diverse genomic data sets were examined to define regulatory miRNA gene networks in DIS and NAFLD of different grades and unique miRNAs targeting LD-associated genes were identified to provide mechanistic information on the onset and progression (micro- versus macrovesicular steatosis) of fatty liver disease. The network analysis revealed 29 out of 56 DIS genes to be commonly regulated by the top 10 regulated miRNAs (Fig. 3a,b) and >70% of all DIS regulated genes (56 over 77 DIS genes) are targeted by these miRNAs. Apart from common DIS regulated genes the transcription factors FOXO1 and FOXA2 and the insulin induced gene 2 are specific targets of miR-335-5p; note polymorphisms of INSIG2 are associated with severe obesity. Furthermore, the patatin-like phospholipase domain-containing protein 3 is a target of miR-335-5p and a single nucleotide polymorphism of this gene is strongly associated with NAFLD of different grades^36^. Other genes regulates by this miRNA are the UDP-Glucose Ceramide Glucosyltransferase (UCGC) and acyl-CoA synthetase long-chain family member 3. Besides, regulation of syntaxin 5 by miR-155-5p is of critical importance. This protein facilitates vesicle docking and its fusion and is part of the SNARE complex. Alike, miR-26b-5p influences translation of caveolin 2 and diacylglycerol O-acyltransferase 1, i.e. a key enzyme in hepatic steatosis in addition to lecithin-cholesterol acyltransferase which catalyzes its esterification for cholesterol transport. For its role in the regulation of the Ah-receptor miR-124-3p is of great importance. Specifically, AhR sustained activation results in induction of fatty acid transport proteins and enhanced lipid uptake via activity of the fatty acid transporter CD36 to eventually cause accumulation of triglycerides leading to hepatic steatosis. Likewise, it is of great significance that AhR suppresses fatty acid oxidation and export of triglycerides from the liver by hampering VLDL synthesis and secretion. Lastly, the AhR engages in a vicious cycle whereby serum triacylglycerides levels are increased through peripheral fat mobilization thus perpetuating hepatic lipid uptake from the systemic circulation^37^.

Regulation of 1-acylglycerol-3-phosphate O-acyltransferase 2 by miR-744-5p is also of key importance. This ER localized protein catalyzes the conversion of lysophosphatidic acid to phosphatidic acid, e.g. a building block in phospholipid synthesis and essential second messenger that mediates cellular functions through different modes of action. Noteworthy is also the regulation of the phosphatase and tensin homolog PTEN by miR21-5p and recent research identified Maf1 as a novel target of PTEN and PI3K signalling to negatively regulate carcinogenesis and lipid metabolism by repressing intracellular lipid accumulation and *de novo* lipogenesis^38^. Alike, this miRNA targets fatty acid synthase and the enzyme catalyze the synthesis of long-chain saturated fatty acids. Among the top 10 miRNAs is let-7b-5p known to influence translation of insulin receptor substrate 2 transcripts and this protein functions as an adaptor to link receptor tyrosine kinases with downstream effectors. Dynein is also regulated by this miRNA and this microtubule-activated ATPase is of uttermost importance in intracellular motility and trafficking of vesicles. A further example is the regulation of the liver receptor homolog 1 (NR5A2) by miR-1 and this nuclear receptor functions as a transcription factor and phospholipid binding protein and plays an essential role in hepatic metabolism. Repression of small heterodimer partner by NR5A2 affects VLDL synthesis and secretion and is deregulated in NAFLD as summarized in the seminal review of Bechmann and colleagues^39^. Lastly, the gene coding for the ER lipid raft associated protein 1 is a target of miR30a-5p and is part of the ERLIN1/ERLIN2 complex to assist ER-associated degradation of inositol 1, 4, 5-trisphosphate receptors (IP3Rs); note, alterations in ER lipid rafts have been shown to contribute to lipotoxicity^40^.

The chosen examples illustrate the benefit in construction molecular circuits based on co-expressed miRNA-gene targets and the knowledge gain by considering mechanistic information regarding common and exclusively regulated genes in DIS and NAFLD. In an effort to define commonalities amongst steatotic drugs four mechanistically linked genes (ELOVL2, ELOVL5, FADS1 and FADS2) were identified as consistently regulated after single and repeated treatments with N = 17 steatotic drugs with FADS1 and FADS2 catalyzing Δ5/Δ6 desaturation of fatty acids to influence arachidonic acid derived eicosanoid, prostaglandins and leukotriene production. The significant regulation of FADS1 and FADS2 will influence arachidonic acid homeostasis and associated herewith immune cell populations with a significant reduction in CD4+/CD3+ T-cell and mononuclear cells as observed in FADS1 null mice and in murine models of NASH^41^. The observed repression of FADS1 and 2 may therefore be consider as an adaption to drug treatment to prevent overt toxicity associated with DIS induced inflammation. While deficiency in unsaturated fatty acids impacts lipid and energy metabolism, membrane structures and signalling pathways, variations in the expression of these enzymes will help to reduce production of inflammatory lipids^42^. Likewise, repression of the fatty acid elongases ELOV2 and 5 denotes coordinate responses in polyunsaturated fatty acid synthesis. Note, overexpression of ELOVL2 enhanced triacylglycerol synthesis in 3T3-L1 and F442A cells and caused induction of the lipogenic genes diacylglycerol acyltransferase-2 and fatty acid-binding protein-4 to support triacylglycerol synthesis and subsequent accumulation of lipid droplets^43^. Modulation of the synthesis of very long-chain fatty acids by ELOV2 and 5 and subsequent desaturations by the Δ5/Δ6-desaturases FADS1 and 2 signifies the relevance of the identified DIS associated genes and assaying mechanistically relevant DIS genomic biomarkers can be easily adapted at the preclinical stage of drug development by merely comparing the hepatic gene expression signature of an experimental drug to DIS and DILI signatures along with appropriate controls.

Testimony to the findings of the present study is also a recently published comprehensive lipidomics approach for an identification of classifier lipid metabolites in urine and plasma and transcriptomic data as identified by RNA-sequencing of liver tissue from a total of N = 31 healthy controls, N = 17 steatotic and N = 20 NASH patients. The transcriptomic data correlated with the clinical stage of disease and signatures of lipids were identified that can be employed as biomarkers for disease staging^44^.

In an effort to define mechanistically relevant serum biomarkers research of the present study identified 24 serum miRNAs as commonly regulated in NASH (Fig. 8). These miRNAs were examined for their role in targeting N = 200 lipid droplet associated genes (supplementary Table S1); a network was constructed whereby 7 of the 24 common regulated miRNAs were involved in the translational control of 41 fatty liver disease specific gene regulations (Fig. 10b). Once again the findings highlight the possibilities by which specific miRNAs instruct pathophysiological processes in fatty liver disease with miR-7-5p targeting suppressor of cytokine signalling SOCs and calveolin-1, i.e. an essential component of membrane lipid rafts with complex functions in cell signalling while the subnetwork of let-7b-5p involves translational control of the LDL receptor, hepatic lipoprotein lipase and SNAP23 that forms a complex with other vesicle-associated membrane protein and is part of the fusion machinery in macro-vesicular steatosis. Further evidence for a coordinate responses in fatty liver disease was obtained by considering regulation of the ER lipid raft associated 2, PPARγ and insulin induced gene 1 by miR-192-5p. The coded proteins are critically involved in lipid and cholesterol homeostasis while inflammatory signalling of the Toll-like receptor 4 and STAT3 pathways was influenced by let-7i-5p and miR-125b-5p, respectively. These and other examples mentioned above clearly demonstrate the benefit of considering mechanistically related miRNA gene target associations in obtaining pathophysiological relevant information by assaying specific serum miRNAs. Indeed, ncRNAs function precisely to repress targets mRNAs and as a result influence expression of proteins and cellular phenotypes^45^. Although a number of miRNAs are widely expressed amongst diverse tissues, certain miRNAs appear to be organ specific. In addition, some miRNAs are conserved across species and hold promise for translational research. To date, over 1800 mature human miRNAs have been identified and recorded in the miRBase registry^46^.

Lastly, the following caveats need to be considered. The study findings are based on the miRTar resource but were not obtained within the same liver tissue used for the DIS study. Nonetheless, the evidence within the miRTar repository is based on independent experimental research and for its high evolutionary conservation the results obtained can be exploited to search for transcriptional regulations of co-expressed microRNA target genes. Specifically, only miRNAs found to be expressed in liver tissue were considered and the research performed focused on the discovery of miRNA targeting genes mechanistically involved in lipid droplet formation and hepatic steatosis. Future research will involve the construction of feed-forward loops (FFLs) to decipher regulatory gene networks as we had demonstrated for the repurposing of drugs in the treatment of cystic fibrosis^47^.

## Conclusions

New insight into co-expressed microRNA target genes in DIS and NAFLD was obtained. An identification of mechanistically linked safety biomarkers will aid drug development and contribute to an understanding of risk for drug induced steatosis and to distinguish amongst other causes of fatty liver disease. Our findings encourage independent validation of the proposed miRNA DIS and NAFLD biomarkers.

## Additional Information

**How to cite this article**: Liu, Z. *et al*. Mechanistically linked serum miRNAs distinguish between drug induced and fatty liver disease of different grades. *Sci. Rep.* **6**, 23709; doi: 10.1038/srep23709 (2016).

## Supplementary Material

Supplementary Information

Supplementary Table S3

Supplementary Table S4

Supplementary Table S5

## Figures and Tables

**Table 1:** Summary information for N = 17 drugs/chemicals which induced hepatic steatosis in rat liver.

Drug/chemical	CAS number	Pathology Image number*	Day	Dose (mg/kg)	Observation
carbon tetrachloride (CCL4)	56-23-5	2663	6 hrs, Day 8, 29	HD_300 mg	clear vacuolar formation
hydroxyzine (HYZ)	68-88-2	19778	Day 29	HD_100 mg	clear vacuolar formation
imipramine (IMI)	50-49-7	33959	Day 29	HD_100 mg	clear vacuolar formation
amitriptyline (AMT)	50-48-6	34287	Day 29	HD_150 mg	clear vacuolar formation
ethinylestradiol (EE)	57-63-6	33709	Day 29	HD_10 mg	clear vacuolar formation
methapyrilene hydrochloride (MP)	91-80-5	46989	Day 29	HD_100 mg	clear vacuolar formation
coumarin (CMA)	91-64-5	29057	Day 29	HD_150 mg	clear vacuolar formation
tetracycline (TC)	60-54-8	11030	9 hr	HD_1000 mg	clear vacuolar formation
lomustine (LS)	13010-47-4	32392	Day 29	HD_6 mg	clear vacuolar formation
vitamin A (VA)	68-26-8	15733	Day 29	HD_100 mg	clear vacuolar formation
diltiazem (DIL)	42399-41-7	22191	Day 15	HD_800 mg	clear vacuolar formation
disulfiram (DSF)	97-77-8	47866	Day 4	HD_600 mg	clear vacuolar formation
colchicine (COL)	64-86-8	41516	24 hr	HD_15 mg	clear vacuolar formation
ethionamide (ETH)	536-33-4	46400	Day 4	HD_300 mg	clear vacuolar formation
ethanol (ETN)	64-17-5	47867	Day 29	HD_4000 mg	clear vacuolar formation
adapin (ADP)	1668-19-5	30832	Day 29	HD_100 mg	clear vacuolar formation
puromycin aminonucleoside (PAN)	58-60-6	55728	Day 8	HD_40 mg	clear vacuolar formation

^*^The image can be retrieved from http://toxico.nibio.go.jp/open-tggates/english/search.html

**Table 2:** Publically available human miRNAs and mRNAs data sets associated with fatty liver disease.

GEO ACESSION number	Species	Diseases	Number of patients	Number of healthy controls	References
*miRNA data sets*					
GSE33857	Homo sapiens (serum)	non-alcoholic steatohepatitis (NASH)	7	12	25
–	Homo sapiens (serum)	non-alcoholic fatty liver disease (NAFLD)	66 benign steatosis	–	22
			94 NASH		
**Total cases**			**167**	**12**	
*mRNA data sets*					
GSE48452	Homo sapiens (tissue)	non-alcoholic steatohepatitis (NASH)	18	14	48
GSE63067	Homo sapiens (tissue)	non-alcoholic steatohepatitis (NASH)	9	7	–
GSE17470	Homo sapiens (tissue)	non-alcoholic steatohepatitis (NASH)	6	4	49
GSE49541	Homo sapiens (tissue)	non-alcoholic fatty liver disease (NAFLD)	40 mild NAFLD	7 control from GSE37031	23
			32 advanced NAFLD		
**Total cases**			**105**	**32**	

**Table 3:** KEGG pathways analysis of DIS genes involved in lipogenesis.

KEGG ID: Pathways	Count	Genes	adjusted *p* values
hsa01040:Biosynthesis of unsaturated fatty acids	4	ELOVL5, FADS1, ELOVL2, FADS2,	7.7E-4
hsa00561:Glycerolipid metabolism	3	DGAT1, AGPAT3, AGPAT2	9.1E-2
hsa00564:Glycerophospholipid metabolism	3	LCAT, AGPAT3, AGPAT2	1.3E-1

**Table 4:** The top 10 ranked drug-induced steatosis regulated miRNAs and their links to NAFLD.

miRNAs	Species	Tissue	Serum	Associated disease	Experimental evidence	References
hsa-miR-335-5p	mouse	liver		lipid metabolism	The up-regulated expressions of miR-335 in liver and white adipose tissue of obese mice might contribute to the pathophysiology of obesity.	27
	rat	liver		hepatic fibrosis	miR-335 restoration inhibited HSC migration, at least in part, via down-regulation the TNC expression, which accounts for the increased numbers of activated HSCs in areas of inflammation during hepatic fibrosis.	50
hsa-miR-124-3p	human	liver		cholestatic liver disease	Down-regulation of miR-124 and up-regulation of miR-200 collaboratively promote bile duct proliferation through the IL-6-STAT3 pathway.	51
	–	MIN6 cells		lipid and glucose process	Over-expression of miR124a leads to exaggerated hormone release under basal conditions and a reduction in glucose-induced secretion.	52
hsa-miR-26b-5p	human	liver		hepatocellular carcinoma (HCC)	The miR-26 expression status is associated with survival and response to adjuvant therapy with interferon-α in liver cancer patients.	53
	rat	liver		NASH	miR-26b was up-regulated with fold change = 8 in differentiating steatohepatitis from steatosis	54
hsa-miR-155-5p	mouse/human	liver		NASH-induced hepatocarcinogenesis	In a microarray analysis conducted on mice fed a choline-deficient L-aminoacid-defined diet, miR-155 was found to be upregulated at early stages of NASH-induced hepatocarcinogenesis, as well as in primary human HCCs compared to matching liver tissues.	55
	human	liver			A gradual ascension of miR-155 expression in cirrhotic and HCC tissues, compared with low expression levels detected in normal liver tissues.	56
	human	liver		Hepatitis C virus - hepatocellular carcinoma	The up-regulated expression of miR-155 induced by inflammation after HCV infection promotes hepatocyte proliferation and tumorigenesis by activating Wnt signalling.	57
	mouse	liver		Hepatic steatosis	Increased expression of miR-155 in models of NAFLD likely plays a critical homeostatic role designed to prevent excessive lipid accumulation in livers that can ultimately lead to liver damage.	58
hsa-miR-30a-5p	zebra fish	liver		hepatic organogenesis	Demonstrated that miR-30a is required for biliary development in hepatic organogenesis in zebra fish.	59
hsa-miR-1	human		Serum	hepatocellular carcinoma	Serum miR-1 is a new independent parameter of OS in HCC patients and may therefore improve the predictive value of classical HCC staging scores.	60
hsa-let-7b-5p	rat	liver		NASH	Let-7b was found to be upregulated in steatohepatitis compared to steatosis samples.	54
hsa-miR-744-5p	human	liver		hepatocellular carcinoma	miR-744 was suggested as an independent predictor of survival in HCC patients after LT and may therefore be a potential biomarker for their prognosis.	61
	human		serum	chronic hepatitis B/NASH	Serum levels of miR-122, -572, -575, -638 and -744 are deregulated in patients with CHB or NASH. The levels of these miRNAs may serve as potential biomarkers for liver injury caused by CHB and NASH.	62
hsa-miR-192-5p	human		serum	drug-induced liver injury	microRNAs (miR-122 and miR-192) are promising biomarkers of acetaminophen-induced acute liver injury (APAP-ALI).	63
	house	liver	serum	drug-induced liver injury	We have demonstrated that specific microRNA species, such as mir-122 and mir-192, both are enriched in the liver tissue and exhibit dose- and exposure duration-dependent changes in the plasma that parallel serum aminotransferase levels and the histopathology of liver degeneration, but their changes can be detected significantly earlier.	64
hsa-miR-21-5p	human		serum	hepatic steatosis	Serum level of miR-21 is higher in the NAFLD patients compared to the control participants.	65
	human	liver		hepatic fibrosis	MicroRNA-21 is important in hepatic fibrosis development, but the mechanism is unclear.	66

**Table 5:** miRNAs in human (A) and animal models (B) of NAFLD of different grades.

miRNAs	Regulation	Species	Liver tissue	Serum	Diseases	Description	References
**(A) A summary of regulated miRNAs in human NAFLD of different grades**							
**Blunt steatosis**							
miR-15b	up-regulated	homo sapiens		serum	hepatic steatosis	The expression of miR-15b was also significantly elevated in the serum of fatty liver disease patients compared with healthy subjects	67
miR-21 miR-34a miR-122 miR-451	up-regulated	homo sapiens		serum	hepatic steatosis	Serum levels of circulating miRNAs, miR-21, miR-34a, miR-122 and miR-451 are associated with nonalcoholic fatty liver disease	65
miR-101	up-regulated	homo sapiens	human THP-1-derived macrophages and HepG2 hepatoblastoma cells		blunt steatosis	miR-101 promotes intracellular cholesterol retention under inflammatory conditions through suppressing ABCA1 expression and suggests that the miR-101-ABCA1 axis may play an intermediary role in the development of NAFLD and vascular atherosclerosis	68
miR-103	up-regulated	homo sapiens		serum	blunt steatosis	Compared with the normal control group, higher serum levels of miR-103 were expressed in the NAFLD group (8.18 ± 0.73 vs 4.23 ± 0.81, P = 0.000).	69
miR-199a-5p	up-regulated	homo sapiens	HepG2 and AML12 cells		hepatic steatosis	Upregulated miR199a-5p in hepatocytes may contribute to impaired FA β-oxidation in mitochondria and aberrant lipid deposits, probably via CAV1 and the PPARα pathway	70
miR-122	down-regulated	homo sapiens	liver tissue		hepatic steatosis	miR-122 is significantly under-expressed in NAFLD subjects.	71
**NASH**							
pri-miR-7-1	up-regulated	homo sapiens	liver tissue		NASH	Histologic NASH correlated positively with the expression levels of pri-miR-16-2 and pri-miR-7-1.	72
pri-miR-16-2							
hsa-miR-125b	down-regulated					Both NASH and ballooning degeneration of hepatocytes correlated negatively with the expression levels of hsa-miR-125b.	
miR-21	down-regulated	homo sapiens		serum	NAFLD	serum levels of miR-21 were lower in patients with NAFLD compared with the healthy controls	73
hsa-miR-28-3p hsa-miR-132 hsa-miR-150 hsa-miR-433 hsa-miR-511 hsa-miR-517a hsa-miR-671		homo sapiens	visceral adipose tissue		NASH	Seven hsa-miR-132, hsa-miR-150, hsa-miR-433, hsa-miR-28-3p, hsa-miR-511, hsa-miR-517a, hsa-miR-671 differentially expressed between NASH patients and non-NASH patients (P < 0.05 after multiple test correction)	74
miR-30c miR-331-3p	up-regulated	homo sapiens		serum	NAFLD	miR-331-3p and miR-30c were differentially expressed between NAFLD and non-NAFLD groups	75
miR-194	down-regulated	homo sapiens	human THP-1 cells		NAFLD	miR-194 negatively regulates the TLR4 signal pathway which is activated by PA through directly negative TRAF6 expression, which is related to inflammatory in NAFLD and obesity	76
miR-296	down-regulated	homo sapiens	liver tissue		NASH	miR-296-5p was reduced in liver samples from nonalcoholic steatohepatitis (NASH) patients compared with patients with simple steatosis (SS) or controls	77
**miRNAs linked to NAFLD and other hepatic disorders**							
miR-16 miR-21 miR-34a miR-122	down-regulated	homo sapiens		serum	chronic hepatitis C and liver fibrosis	Serum levels of miR-34a and miR-122 may represent novel, noninvasive biomarkers of diagnosis and histological disease severity in patients with CHC or NAFLD	78
hsa-miR-17 hsa-miR-186 hsa-miR-219a-2 hsa-miR-373 hsa-miR-376b hsa-miR-378c hsa-miR-378i hsa-miR-590 hsa-miR-1286 hsa-miR-3611 hsa-miR-5699	down-regulated	homo sapiens	liver tissue		blunt steatosis/fibrosis or cirrhosis	A total of 75 miRNAs showing statistically significant evidence for differential expression between the NAFLD VS. Non-NAFLD, including 30 upregulated and 45 downregulated miRNAs	79
hsa-miR-31 hsa-miR-92b hsa-miR-150 hsa-miR-182 hsa-miR-183 hsa-miR-200a hsa-miR-224 hsa-miR-708 hsa-miR-766 hsa-miR-3613	up-regulated						
hsa-miR-27b-3p hsa-miR-122-5p hsa-miR-192-5p hsa-miR-1290	up-regulated	homo sapiens		serum	blunt steatosis/NASH	a serum microRNA panel (hsa-miR-122-5p, hsa-miR-1290, hsa-miR-27b-3p, and hsa-miR-192-5p) with considerable clinical value in NAFLD diagnosis	80
miR-33a miR-224	up-regulated	homo sapiens	liver tissue		steatotic chronic hepatitis C	he expression of miR-33a and miR-224 were elevated in CHC-Steatosis and Steatosis in comparison to control tissue (P < 0.01)	81
miR-122	up-regulated	homo sapiens		serum	hepatic steatosis and fibrosis	The hepatic and serum miR-122 levels were associated with hepatic steatosis and fibrosis. The serum miR-122 level can be a useful predictive marker of liver fibrosis in patients with NAFLD	82
**(B) A summary of regulated miRNAs in animal models of NAFLD of different grades**							
**Blunt steatosis**							
miR-10b	down-regulated		L02 cells		hepatic steatosis	The established miRNA profile of the steatotic L02 cell model and the novel effect of miRNA-10b in regulating hepatocyte steatosis may provide a new explanation of the pathogenesis of NAFLD	83
miR-21	down-regulated	C57BL/6J mice	liver tissue		hepatic steatosis	miR-21 expression was decreased in livers from high-fat diet-fed mice and Hepa 1-6 cells treated with SA.	84
miR-27 miR-122 miR-451	down-regulated	rat	liver tissue		hepatic steatosis	The miRNAs analysis showed the significant down regulation of three miRNAs (miR-122, miR-451 and miR-27) and the up regulation of miR-200a, miR-200b and miR-429 in HFD, SD-HF and HFD-HF rats.	85
miR-200a miR-200b miR-429	up-regulated						
miR-34a miR-122 miR-181a miR-192 miR-200b	up-regulated	Mouse		serum	hepatic steatosis	The levels of circulating miR-34a, miR-122, miR-181a, miR-192, and miR-200b miRNAs were significantly correlated with a severity of NAFLD-specific liver pathomorphological features, with the strongest correlation occurring with miR-34a.	86
miR-34a	up-regulated	mouse	ob/ob mouse liver		hepatic steatosis	Up regulation of miR-34a and down regulation of miR-122 was found in livers of STZ-induced diabetic mice.	87
miR-122	down-regulated						
miR-122	up-regulated	rat		serum	blunt steatosis	serum miR-122 level is indeed useful for assessing early NAFLD and might be superior to clinical markers traditionally used to monitor hepatic disease	88
miR-122 miR-192	up-regulated	mouse		serum	blunt steatosis	the liver appears to be an important source of circulating EVs in NAFLD animals as evidenced by the enrichment in blood with miR-122 and 192	89
miR-125b	up-regulated	mouse	primary mouse hepatocytes		blunt steatosis	Estrogen protects against hepatic steatosis in female mice via up-regulating miR-125b expression	90
miR-146a miR-146b miR-152 miR-200a miR-200b miR-200c	up-regulated	rat	HepG2 cells and human hepatocytes		blunt steatosis	miR-200a, miR-200b, miR-200c, miR-146a, miR-146b and miR-152 were up-regulated both *in vitro* and *vivo*.	91
miR-155	up-regulated	mouse	liver tissue		lipid metabolism	miR-155 gain of function the altered lipid metabolism and provide new insights into the metabolic state of the liver in Rm155LG/Alb-Cre mice	92
miR-155 miR-200b	up-regulated	rat	NAFLD rats and in the free fatty acid-treated HepG2		blunt steatosis	The pharmacological inhibition of EZH2 by 3-Deazaneplanocin A (DZNep) significantly reduces EZH2 expression/activity, while it increases lipid accumulation, inflammatory molecules and miR-200b/155	93
miR-302a	down-regulated	mouse	liver tissue		hepatic steatosis	miR-302a may prove to be a valuable therapeutic target in the regulation of hepatic fatty acid utilization and insulin resistance.	94
miR-467b	down-regulated	mouse	liver tissue		hepatic steatosis	Down regulation of miR-467b is involved in the development of hepatic steatosis by modulating the expression of its target, LPL.	95
**NASH**							
miR-21	up-regulated	mouse	liver tissue		NASH	The studies show the novel role of leptin-NADPH oxidase induction of miR21 as a key regulator of TGF-β signalling and fibrogenesis in experimental and human NASH	96
miR-34a	up-regulated	rat	liver tissue		NASH	A link between liver cell apoptosis and miR-34a/SIRT1/p53 signalling, specifically modulated by UDCA, and NAFLD severity.	97
miR-122	down-regulated	mouse	HCC tissue		NASH	Silencing of miR-122 is an early event during hepatocarcinogenesis from NASH, and that miR-122 could be a novel molecular marker for evaluating the risk of HCC in patients with NASH	98
miR-122	down-regulated	mouse	liver and hepatocyte		NASH	Decreased liver miR-122 contributes to up regulation of modulators of tissue remodelling (HIF-1α, vimentin and MAP3K3) and might play a role in NASH-induced liver fibrosis	99
miR-122	up-regulated	mouse		serum	NASH	Serum levels of miR-122 can potentially be used as a sensitive biomarker for the early detection of hepatotoxicity and can aid in monitoring the extent of NAFLD-associated liver injury in mouse efficacy models	100
miR-199a-5p miR-219-5p		mouse	liver tissue		obesity-induced steato-hepatitis	RvD1 acts as a facilitator of the hepatic resolution process by reducing the inflammatory component of obesity-induced NASH, which was regulated by miR-219-5p and miR-199a-5p	101
miR-199a-5p	up-regulated	mouse	C57BL/6J mice		NASH	miR-199a-5p plays a key role in the progression of NASH through inhibition of NCOR1 translation, and provide novel insights into NASH pathogenesis	102
miR-451	down-regulated	mouse	Liver tissue		NASH	The negative regulation of miR-451 in fatty acid-induced inflammation via the AMPK/AKT pathway and demonstrate potential therapeutic applications for miR-451 in preventing the progression from simple steatosis to severely advanced liver disease	103
**miRNAs linked to NAFLD and other hepatic disorders**							
miR-24	up-regulated	mouse	liver tissue		hepatic lipid accumulation and hyperlipidemia	miR-24 promotes hepatic lipid accumulation and hyperlipidemia by repressing Insig1, and suggest the use of miR-24 inhibitor as a potential therapeutic agent for NAFLD and/or atherosclerosis	104
miR-34a	down-regulated	mouse	Liver tissue		Hepatic ischemia/reperfusion (I/R) injury	The miR-34a/SIRT1 pathway may represent a therapeutic target for hepatic injury.	105
miR-200a miR-223	up-regulated	mouse	male A/J, 129S1/SvImJ and WSB/EiJ mice		liver fibrosis	Downregulation of IRP1 was linked to an increased expression of microRNAs miR-200a and miR-223, which was negatively correlated with IRP1. The results of this study demonstrate that the interstrain variability in the extent of fibrogenesis was associated with a strain-dependent deregulation of hepatic iron homeostasis.	106
