# Supplementary material for: Mechanistically linked serum miRNAs distinguish between drug induced and fatty liver disease of different grades
Source: Sci Rep. 2016 Apr 5;6:23709. doi: 10.1038/srep23709 (PMC4820692; doi:10.1038/srep23709)
Supplement: Supplementary Information [file srep23709-s1.pdf]

SUPPORTING INFORMATION

Mechanistically linked serum miRNAs distinguish between drug induced and fatty liver disease of different grades

Zhichao Liu[1#], Yuping Wang[1#], Jürgen Borlak[2*] and Weida Tong[1*]

[1] Division of Bioinformatics and Biostatistics, National Center for Toxicological Research, U.S. Food and Drug Administration, Jefferson, Arkansas, USA

[2] Centre for Pharmacology and Toxicology, Hannover Medical School, Hannover, Germany

[#] Equal contributors

SUPPLEMENTARY FIGURE AND TABLE LEGENDS

Supplementary Fig. S1. Venn diagram of NAFLD regulated miRNAs based on computational prediction and miRTarBase queries

Supplementary Table S1. List of 200 genes considered to be mechanistically relevant in the process of lipid droplet biogenesis in hepatocytes as recently published by us [17].

Supplementary Table S2. Summary of the 409 co-expressed miRNA-gene targets in DIS and NAFLD.

Supplementary Table S3. Drug, dose and time associations of co-expressed miRNA- gene targets in DIS.

Supplementary Table S4. STRING analysis of protein-protein interactions after single and repeated drug treatment of rats with 17 steatotic drugs.

Supplementary Table S5. Results of 12 different computational algorithms to predicted miRNAs involved in DIS regulated genes.

Fig. S1

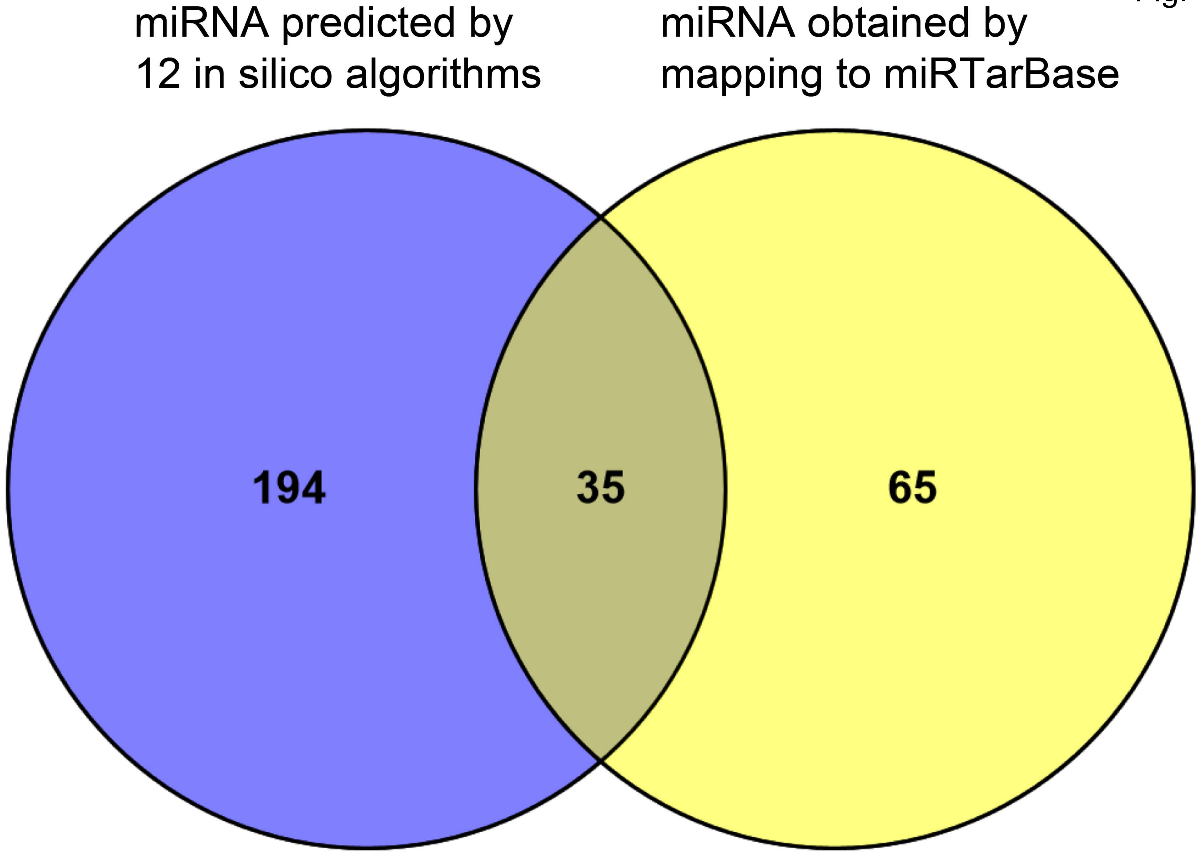
miRNA predicted by
12 in silico algorithms
miRNA obtained by
mapping to miRTarBase
194
35
65

Supplementary Table S1

Gene Symbols	Related Mechanism
MTORC1	Lipogenesis
MTORC2	Lipogenesis
PPAP2	Lipogenesis
ACC1	Lipogenesis
ACAT1	Lipogenesis
ACAT2	Lipogenesis
ACLY	Lipogenesis
ANXA2	Lipogenesis
ANXA5	Lipogenesis
ANXA6	Lipogenesis
FAS	Lipogenesis
CNR1	Lipogenesis
INSIG1	Lipogenesis
LCAT	Lipogenesis
FADS1	Lipogenesis
PPARG	Lipogenesis
RHEB	Lipogenesis
SCD1	Lipogenesis
SREBF1	Lipogenesis
TSC1	Lipogenesis
TSC2	Lipogenesis
UGCG	Lipogenesis
DHAP-AT	Lipogenesis
AGPS	Lipogenesis
DGAT1	Lipogenesis
FADS2	Lipogenesis
AGPAT1	Lipogenesis
AGPAT2	Lipogenesis
SPT1	Lipogenesis
SCAP	Lipogenesis
MLXIPL	Lipogenesis
INSIG2	Lipogenesis
ELOVL2	Lipogenesis
AGPAT5	Lipogenesis
AGPAT3	Lipogenesis
NCEH1	Lipogenesis
GPAT1	Lipogenesis
ELOVL5	Lipogenesis
ELOVL6	Lipogenesis
DGAT2	Lipogenesis
CREB3L3	Lipogenesis
GPAT3	Lipogenesis
GPAT2	Lipogenesis
S6K	Lipogenesis
ELOVL3	Lipogenesis

CPT1	Fatty acid oxidation
CPT2	Fatty acid oxidation
ACSL1	Fatty acid oxidation
FOXA2	Fatty acid oxidation
MTP	Fatty acid oxidation
PPARA	Fatty acid oxidation
PPARB	Fatty acid oxidation
FAF2	Fatty acid oxidation
DERL1	Fatty acid oxidation
ACSL3	Fatty acid oxidation
PI3KC2G	Lipid transport
THR	Lipid transport
ABCA1	Lipid transport
AHR	Lipid transport
AKT1	Lipid transport
AKT2	Lipid transport
CD36	Lipid transport
FABP1	Lipid transport
FABP6	Lipid transport
FABP7	Lipid transport
NR5A2	Lipid transport
LDLR	Lipid transport
RXRA	Lipid transport
RXRB	Lipid transport
RXRG	Lipid transport
NR1I2	Lipid transport
FXR	Lipid transport
NR1H3	Lipid transport
FATP5	Lipid transport
SLC27A5	Lipid transport
FATP2	Lipid transport
GSTA	Bio-markers
LAMA	Bio-markers
LAMB	Bio-markers
LAMC	Bio-markers
TXN1	Bio-markers
COL4A4	Bio-markers
COL4A6	Bio-markers
CRP	Bio-markers
GGT	Bio-markers
GLUD1	Bio-markers
GOT1	Bio-markers
GPT	Bio-markers
KRT18	Bio-markers
LDH	Bio-markers
TIMP1	Bio-markers
TXN2	Bio-markers
FGF21	Bio-markers

MIR122	Bio-markers
DNAHC	LD proteins
KIF	LD proteins
PKA	LD proteins
RAB11	LD proteins
SEC22	LD proteins
PLIN2	LD proteins
APOB	LD proteins
APOE	LD proteins
ARG1	LD proteins
CAV1	LD proteins
CAV2	LD proteins
CES1	LD proteins
DYNC1H1	LD proteins
DYNC1I1	LD proteins
PLD	LD proteins
LIPE	LD proteins
LPL	LD proteins
NSF	LD proteins
PLIN1	LD proteins
PKD	LD proteins
RAB5	LD proteins
SNCG	LD proteins
STX5	LD proteins
VIM	LD proteins
RAB7	LD proteins
DYNLL1	LD proteins
VAMP4	LD proteins
SNAP23	LD proteins
NAPA	LD proteins
ATG5	LD proteins
FLOT1	LD proteins
PLIN3	LD proteins
VTI1B	LD proteins
ATG7	LD proteins
ERLIN1	LD proteins
RAB32	LD proteins
ERLIN2	LD proteins
MGLL	LD proteins
RAB18	LD proteins
HILPDA	LD proteins
ABHD5	LD proteins
ARFGAP1	LD proteins
PNPLA2	LD proteins
CIDEC	LD proteins
DYNC2H1	LD proteins
PNPLA3	LD proteins
MAP1LC3B	LD proteins

DNAAF1	LD proteins
FITM2	LD proteins
FITM1	LD proteins
PLIN5	LD proteins
SEC1	LD proteins
PLIN4	LD proteins
LY6C	Signalling events
EIF-2ALPHA	Signalling events
CXCL2	Signalling events
IL1	Signalling events
IL6	Signalling events
IL8	Signalling events
IL10	Signalling events
IL12	Signalling events
IL17	Signalling events
IL18	Signalling events
NFKB1	Signalling events
NOS2	Signalling events
SERPINE1	Signalling events
PRKAA1	Signalling events
PRKAA2	Signalling events
PRKAB1	Signalling events
PRKAB2	Signalling events
PRKAG1	Signalling events
MAPK2	Signalling events
MAPK1	Signalling events
MAPK8	Signalling events
MAPK9	Signalling events
PTEN	Signalling events
PTGER2	Signalling events
NFKB3	Signalling events
ROCK1	Signalling events
CCL2	Signalling events
STAT3	Signalling events
STAT5	Signalling events
TGFB1	Signalling events
TGFB2	Signalling events
TLR4	Signalling events
TNFA	Signalling events
SOCS1	Signalling events
EIF-2GAMMA	Signalling events
SOCS2	Signalling events
EIF-2BETA	Signalling events
SOCS3	Signalling events
ROCK2	Signalling events
USP20	Signalling events
USP33	Signalling events
PRKAG2	Signalling events

PRKAG3	Signalling events
CCR2	Signalling events
CEBPA	Glucose metabolism
FOXO1	Glucose metabolism
GK	Glucose metabolism
HNF4A	Glucose metabolism
IRS1	Glucose metabolism
PKLR	Glucose metabolism
SLC2A1	Glucose metabolism
IRS2	Glucose metabolism
ANGPTL4	Glucose metabolism

Supplementary Table S2

DIS genes	miRNAs in miRTarBase	Mechanism
IL18	hsa-miR-346	Signalling events
IL6	hsa-let-7a-5p	Signalling events
IL6	hsa-miR-1	Signalling events
IL6	hsa-miR-124-3p	Signalling events
IL6	hsa-miR-155-5p	Signalling events
IL6	hsa-miR-26a-5p	Signalling events
IL6	hsa-miR-335-5p	Signalling events
IL6	hsa-miR-365a-3p	Signalling events
IL6	hsa-miR-98-5p	Signalling events
MAPK1	hsa-let-7b-5p	Signalling events
MAPK1	hsa-miR-15b-5p	Signalling events
MAPK1	hsa-miR-199a-3p	Signalling events
MAPK1	hsa-miR-28-5p	Signalling events
MAPK1	hsa-miR-30a-5p	Signalling events
MAPK1	hsa-miR-320a	Signalling events
MAPK1	hsa-miR-335-5p	Signalling events
MAPK1	hsa-miR-766-3p	Signalling events
MAPK1	hsa-miR-769-5p	Signalling events
MAPK1	hsa-miR-92b-3p	Signalling events
MAPK9	hsa-miR-106b-5p	Signalling events
MAPK9	hsa-miR-141-3p	Signalling events
MAPK9	hsa-miR-17-5p	Signalling events
MAPK9	hsa-miR-192-5p	Signalling events
MAPK9	hsa-miR-199a-3p	Signalling events
MAPK9	hsa-miR-20a-5p	Signalling events
MAPK9	hsa-miR-215-5p	Signalling events
MAPK9	hsa-miR-27b-3p	Signalling events
MAPK9	hsa-miR-7-5p	Signalling events
MAPK9	hsa-miR-92a-3p	Signalling events
MAPK9	hsa-miR-93-5p	Signalling events
NFKB1	hsa-let-7a-5p	Signalling events
NFKB1	hsa-miR-146a-5p	Signalling events
NFKB1	hsa-miR-146b-5p	Signalling events
NFKB1	hsa-miR-155-5p	Signalling events
NFKB1	hsa-miR-15a-5p	Signalling events
NFKB1	hsa-miR-16-5p	Signalling events
NFKB1	hsa-miR-21-5p	Signalling events
NFKB1	hsa-miR-26b-5p	Signalling events
NFKB1	hsa-miR-9-5p	Signalling events
NFKB1	hsa-miR-92a-3p	Signalling events
NFKB1	mmu-miR-210-3p	Signalling events
PRKAA1	hsa-miR-128-3p	Signalling events
PRKAA1	hsa-miR-130b-3p	Signalling events
PRKAA1	hsa-miR-148b-3p	Signalling events
PRKAA1	hsa-miR-19b-3p	Signalling events
PRKAA1	hsa-miR-29b-3p	Signalling events
PRKAA1	hsa-miR-30a-5p	Signalling events
PRKAA1	hsa-miR-30e-5p	Signalling events
PRKAA1	hsa-miR-652-3p	Signalling events
PRKAA2	hsa-let-7b-5p	Signalling events
PRKAA2	hsa-miR-182-5p	Signalling events
PRKAA2	hsa-miR-192-5p	Signalling events
PRKAA2	hsa-miR-98-5p	Signalling events
PRKAB1	hsa-miR-122-5p	Signalling events
PTEN	hsa-miR-103a-3p	Signalling events
PTEN	hsa-miR-106b-5p	Signalling events
PTEN	hsa-miR-107	Signalling events
PTEN	hsa-miR-141-3p	Signalling events
PTEN	hsa-miR-144-3p	Signalling events
PTEN	hsa-miR-17-5p	Signalling events
PTEN	hsa-miR-181b-5p	Signalling events
PTEN	hsa-miR-18a-5p	Signalling events
PTEN	hsa-miR-193b-3p	Signalling events
PTEN	hsa-miR-19a-3p	Signalling events
PTEN	hsa-miR-19b-3p	Signalling events
PTEN	hsa-miR-20a-5p	Signalling events
PTEN	hsa-miR-21-5p	Signalling events
PTEN	hsa-miR-214-3p	Signalling events
PTEN	hsa-miR-216a-5p	Signalling events
PTEN	hsa-miR-217	Signalling events

77 unique genes	157 unique miRNAs
ABCA1	hsa-let-7a-5p
ACAT1	hsa-let-7b-5p
ACLY	hsa-let-7e-5p
ACSL1	hsa-let-7i-5p
ACSL3	hsa-miR-1
AGPAT2	hsa-miR-100-5p
AGPAT3	hsa-miR-103a-3p
AHR	hsa-miR-106a-5p
AKT1	hsa-miR-106b-3p
AKT2	hsa-miR-106b-5p
ANGPTL4	hsa-miR-107
ANXA2	hsa-miR-10a-3p
ANXA5	hsa-miR-10a-5p
ANXA6	hsa-miR-10b-5p
ARFGAP1	hsa-miR-122-5p
ARG1	hsa-miR-1226-3p
ATG7	hsa-miR-1229-3p
CAV1	hsa-miR-124-3p
CAV2	hsa-miR-125b-5p
CEBPA	hsa-miR-126-3p
CPT2	hsa-miR-1260b
DERL1	hsa-miR-128-3p
DGAT1	hsa-miR-1280
DYNC1H1	hsa-miR-1296-5p
DYNC2H1	hsa-miR-1301-3p
ELOVL2	hsa-miR-130a-3p
ELOVL5	hsa-miR-130b-3p
ERLIN1	hsa-miR-132-3p
FABP7	hsa-miR-138-5p
FADS1	hsa-miR-141-3p
FADS2	hsa-miR-142-3p
FAS	hsa-miR-143-3p
FITM2	hsa-miR-144-3p
FLOT1	hsa-miR-145-5p
FOXA2	hsa-miR-146a-5p
FOXO1	hsa-miR-146b-5p
GK	hsa-miR-148a-3p
GLUD1	hsa-miR-148b-3p
GOT1	hsa-miR-149-3p
HNF4A	hsa-miR-149-5p
IL18	hsa-miR-152-3p
IL6	hsa-miR-153-3p
INSIG2	hsa-miR-155-5p
IRS1	hsa-miR-15a-5p
IRS2	hsa-miR-15b-5p
LCAT	hsa-miR-16-5p
LDLR	hsa-miR-17-3p
MAPK1	hsa-miR-17-5p
MAPK9	hsa-miR-181b-5p
MGLL	hsa-miR-182-5p
NFKB1	hsa-miR-183-5p
NR1H3	hsa-miR-184
NR1I2	hsa-miR-185-5p
NR5A2	hsa-miR-186-5p
NSF	hsa-miR-18a-3p
PNPLA3	hsa-miR-18a-5p
PPARA	hsa-miR-191-5p
PPARG	hsa-miR-192-5p
PRKAA1	hsa-miR-193a-5p
PRKAA2	hsa-miR-193b-3p
PRKAB1	hsa-miR-194-5p
PTEN	hsa-miR-196a-5p
RAB32	hsa-miR-197-3p
ROCK1	hsa-miR-199a-3p
ROCK2	hsa-miR-199a-5p
SCAP	hsa-miR-19a-3p
SNAP23	hsa-miR-19b-3p
SOCS2	hsa-miR-203a
SOCS3	hsa-miR-20a-5p
SREBF1	hsa-miR-20b-5p

PTEN	hsa-miR-221-3p	Signalling events
PTEN	hsa-miR-222-3p	Signalling events
PTEN	hsa-miR-23a-3p	Signalling events
PTEN	hsa-miR-23b-3p	Signalling events
PTEN	hsa-miR-26a-5p	Signalling events
PTEN	hsa-miR-29a-3p	Signalling events
PTEN	hsa-miR-29b-3p	Signalling events
PTEN	hsa-miR-494-3p	Signalling events
PTEN	hsa-miR-519a-3p	Signalling events
PTEN	hsa-miR-519c-3p	Signalling events
PTEN	hsa-miR-519d-3p	Signalling events
PTEN	hsa-miR-93-5p	Signalling events
ROCK1	hsa-miR-1280	Signalling events
ROCK1	hsa-miR-146a-5p	Signalling events
ROCK1	hsa-miR-148b-3p	Signalling events
ROCK1	hsa-miR-584-5p	Signalling events
ROCK2	hsa-miR-124-3p	Signalling events
ROCK2	hsa-miR-138-5p	Signalling events
SOCS2	hsa-miR-16-5p	Signalling events
SOCS2	hsa-miR-194-5p	Signalling events
SOCS2	hsa-miR-7-5p	Signalling events
SOCS3	hsa-miR-203a	Signalling events
SOCS3	hsa-miR-30c-5p	Signalling events
STAT3	hsa-let-7e-5p	Signalling events
STAT3	hsa-miR-125b-5p	Signalling events
STAT3	hsa-miR-155-5p	Signalling events
STAT3	hsa-miR-20a-5p	Signalling events
STAT3	hsa-miR-20b-5p	Signalling events
STAT3	hsa-miR-21-5p	Signalling events
STAT3	hsa-miR-337-3p	Signalling events
STAT3	hsa-miR-92a-3p	Signalling events
STAT3	hsa-miR-93-5p	Signalling events
TGFB1	hsa-miR-21-5p	Signalling events
TGFB1	hsa-miR-24-3p	Signalling events
TGFB1	hsa-miR-633	Signalling events
TGFB1	hsa-miR-744-5p	Signalling events
TLR4	hsa-let-7i-5p	Signalling events
TLR4	hsa-miR-1	Signalling events
TLR4	hsa-miR-146a-5p	Signalling events
TLR4	hsa-miR-21-5p	Signalling events
TLR4	hsa-miR-335-5p	Signalling events
ACAT1	hsa-miR-1260b	Lipogenesis
ACAT1	hsa-miR-21-5p	Lipogenesis
ACAT1	hsa-miR-23b-3p	Lipogenesis
ACLY	hsa-miR-10a-3p	Lipogenesis
ACLY	hsa-miR-10b-5p	Lipogenesis
ACLY	hsa-miR-1229-3p	Lipogenesis
ACLY	hsa-miR-125b-5p	Lipogenesis
ACLY	hsa-miR-149-5p	Lipogenesis
ACLY	hsa-miR-18a-3p	Lipogenesis
ACLY	hsa-miR-320b	Lipogenesis
ACLY	hsa-miR-324-3p	Lipogenesis
ACLY	hsa-miR-361-5p	Lipogenesis
ACLY	hsa-miR-484	Lipogenesis
ACLY	hsa-miR-769-3p	Lipogenesis
ACLY	hsa-miR-877-3p	Lipogenesis
ACLY	hsa-miR-92a-3p	Lipogenesis
ACLY	hsa-miR-93-3p	Lipogenesis
AGPAT2	hsa-miR-744-5p	Lipogenesis
AGPAT3	hsa-miR-100-5p	Lipogenesis
AGPAT3	hsa-miR-149-5p	Lipogenesis
AGPAT3	hsa-miR-18a-3p	Lipogenesis
AGPAT3	hsa-miR-24-3p	Lipogenesis
AGPAT3	hsa-miR-26b-5p	Lipogenesis
AGPAT3	hsa-miR-335-5p	Lipogenesis
AGPAT3	hsa-miR-744-5p	Lipogenesis
ANXA2	hsa-miR-1	Lipogenesis
ANXA2	hsa-miR-132-3p	Lipogenesis
ANXA2	hsa-miR-155-5p	Lipogenesis
ANXA2	hsa-miR-769-3p	Lipogenesis
ANXA5	hsa-miR-124-3p	Lipogenesis
ANXA5	hsa-miR-26b-5p	Lipogenesis
ANXA5	hsa-miR-330-5p	Lipogenesis

STAT3	hsa-miR-21-5p
STX5	hsa-miR-214-3p
TGFB1	hsa-miR-215-5p
TLR4	hsa-miR-216a-5p
TSC2	hsa-miR-217
UGCG	hsa-miR-22-3p
VIM	hsa-miR-221-3p
	hsa-miR-222-3p
	hsa-miR-223-3p
	hsa-miR-23a-3p
	hsa-miR-23b-3p
	hsa-miR-24-3p
	hsa-miR-25-3p
	hsa-miR-26a-5p
	hsa-miR-26b-3p
	hsa-miR-26b-5p
	hsa-miR-27a-3p
	hsa-miR-27b-3p
	hsa-miR-28-5p
	hsa-miR-296-3p
	hsa-miR-29a-3p
	hsa-miR-29b-3p
	hsa-miR-302a-3p
	hsa-miR-302b-3p
	hsa-miR-302c-3p
	hsa-miR-302d-3p
	hsa-miR-30a-5p
	hsa-miR-30c-5p
	hsa-miR-30e-5p
	hsa-miR-320a
	hsa-miR-320b
	hsa-miR-320c
	hsa-miR-324-3p
	hsa-miR-324-5p
	hsa-miR-330-5p
	hsa-miR-331-3p
	hsa-miR-335-5p
	hsa-miR-337-3p
	hsa-miR-33a-5p
	hsa-miR-33b-5p
	hsa-miR-340-5p
	hsa-miR-346
	hsa-miR-34a-5p
	hsa-miR-34b-5p
	hsa-miR-34c-5p
	hsa-miR-3605-3p
	hsa-miR-361-5p
	hsa-miR-365a-3p
	hsa-miR-373-3p
	hsa-miR-374b-5p
	hsa-miR-375
	hsa-miR-378a-3p
	hsa-miR-423-3p
	hsa-miR-449a
	hsa-miR-451a
	hsa-miR-455-3p
	hsa-miR-484
	hsa-miR-494-3p
	hsa-miR-500a-3p
	hsa-miR-504-5p
	hsa-miR-519a-3p
	hsa-miR-519c-3p
	hsa-miR-519d-3p
	hsa-miR-584-5p
	hsa-miR-613
	hsa-miR-615-3p
	hsa-miR-625-5p
	hsa-miR-629-5p
	hsa-miR-633
	hsa-miR-652-3p
	hsa-miR-7-5p
	hsa-miR-708-5p
	hsa-miR-744-5p

ANXA6	hsa-miR-124-3p	Lipogenesis	hsa-miR-765
ANXA6	hsa-miR-324-5p	Lipogenesis	hsa-miR-766-3p
ANXA6	hsa-miR-877-3p	Lipogenesis	hsa-miR-769-3p
DGAT1	hsa-miR-125b-5p	Lipogenesis	hsa-miR-769-5p
DGAT1	hsa-miR-183-5p	Lipogenesis	hsa-miR-877-3p
DGAT1	hsa-miR-26b-5p	Lipogenesis	hsa-miR-9-5p
DGAT1	hsa-miR-484	Lipogenesis	hsa-miR-92a-3p
ELOVL2	hsa-miR-10a-5p	Lipogenesis	hsa-miR-92b-3p
ELOVL2	hsa-miR-17-3p	Lipogenesis	hsa-miR-93-3p
ELOVL2	hsa-miR-26b-5p	Lipogenesis	hsa-miR-93-5p
ELOVL5	hsa-miR-124-3p	Lipogenesis	hsa-miR-96-5p
ELOVL5	hsa-miR-16-5p	Lipogenesis	hsa-miR-98-5p
ELOVL5	hsa-miR-192-5p	Lipogenesis	hsa-miR-99a-5p
ELOVL5	hsa-miR-19b-3p	Lipogenesis	hsa-miR-210-3p
ELOVL5	hsa-miR-215-5p	Lipogenesis	
ELOVL5	hsa-miR-30a-5p	Lipogenesis	
ELOVL5	hsa-miR-324-5p	Lipogenesis	
FADS1	hsa-miR-1	Lipogenesis	
FADS1	hsa-miR-155-5p	Lipogenesis	
FADS1	hsa-miR-192-5p	Lipogenesis	
FADS1	hsa-miR-215-5p	Lipogenesis	
FADS1	hsa-miR-335-5p	Lipogenesis	
FADS2	hsa-let-7b-5p	Lipogenesis	
FADS2	hsa-miR-18a-3p	Lipogenesis	
FADS2	hsa-miR-26b-5p	Lipogenesis	
FADS2	hsa-miR-324-3p	Lipogenesis	
FADS2	hsa-miR-335-5p	Lipogenesis	
FADS2	hsa-miR-423-3p	Lipogenesis	
FADS2	hsa-miR-500a-3p	Lipogenesis	
FAS	hsa-miR-106a-5p	Lipogenesis	
FAS	hsa-miR-146a-5p	Lipogenesis	
FAS	hsa-miR-21-5p	Lipogenesis	
FAS	hsa-miR-504-5p	Lipogenesis	
FAS	hsa-miR-98-5p	Lipogenesis	
INSIG2	hsa-miR-142-3p	Lipogenesis	
INSIG2	hsa-miR-335-5p	Lipogenesis	
INSIG2	hsa-miR-373-3p	Lipogenesis	
INSIG2	hsa-miR-375	Lipogenesis	
LCAT	hsa-miR-26b-5p	Lipogenesis	
PPARG	hsa-miR-1	Lipogenesis	
PPARG	hsa-miR-130a-3p	Lipogenesis	
PPARG	hsa-miR-130b-3p	Lipogenesis	
PPARG	hsa-miR-138-5p	Lipogenesis	
PPARG	hsa-miR-192-5p	Lipogenesis	
PPARG	hsa-miR-20b-5p	Lipogenesis	
PPARG	hsa-miR-215-5p	Lipogenesis	
PPARG	hsa-miR-27b-3p	Lipogenesis	
SCAP	hsa-miR-10a-5p	Lipogenesis	
SCAP	hsa-miR-181b-5p	Lipogenesis	
SCAP	hsa-miR-484	Lipogenesis	
SCAP	hsa-miR-93-3p	Lipogenesis	
SREBF1	hsa-let-7e-5p	Lipogenesis	
SREBF1	hsa-miR-10a-5p	Lipogenesis	
SREBF1	hsa-miR-185-5p	Lipogenesis	
SREBF1	hsa-miR-25-3p	Lipogenesis	
SREBF1	hsa-miR-335-5p	Lipogenesis	
SREBF1	hsa-miR-378a-3p	Lipogenesis	
SREBF1	hsa-miR-484	Lipogenesis	
SREBF1	hsa-miR-744-5p	Lipogenesis	
SREBF1	hsa-miR-93-3p	Lipogenesis	
TSC2	hsa-miR-17-5p	Lipogenesis	
TSC2	hsa-miR-296-3p	Lipogenesis	
TSC2	hsa-miR-744-5p	Lipogenesis	
UGCG	hsa-miR-335-5p	Lipogenesis	
UGCG	hsa-miR-375	Lipogenesis	
ABCA1	hsa-miR-19a-3p	Lipid transport	
ABCA1	hsa-miR-26a-5p	Lipid transport	
ABCA1	hsa-miR-26b-5p	Lipid transport	
ABCA1	hsa-miR-27a-3p	Lipid transport	
ABCA1	hsa-miR-33a-5p	Lipid transport	
ABCA1	hsa-miR-33b-5p	Lipid transport	
AHR	hsa-miR-124-3p	Lipid transport	
AHR	hsa-miR-130b-3p	Lipid transport	

AHR	hsa-miR-26a-5p	Lipid transport
AHR	hsa-miR-625-5p	Lipid transport
AHR	hsa-miR-98-5p	Lipid transport
AKT1	hsa-miR-125b-5p	Lipid transport
AKT1	hsa-miR-143-3p	Lipid transport
AKT1	hsa-miR-149-3p	Lipid transport
AKT1	hsa-miR-185-5p	Lipid transport
AKT1	hsa-miR-193b-3p	Lipid transport
AKT1	hsa-miR-26b-5p	Lipid transport
AKT1	hsa-miR-302a-3p	Lipid transport
AKT1	hsa-miR-302b-3p	Lipid transport
AKT1	hsa-miR-302c-3p	Lipid transport
AKT1	hsa-miR-302d-3p	Lipid transport
AKT1	hsa-miR-451a	Lipid transport
AKT2	hsa-miR-124-3p	Lipid transport
AKT2	hsa-miR-1296-5p	Lipid transport
AKT2	hsa-miR-149-5p	Lipid transport
AKT2	hsa-miR-184	Lipid transport
AKT2	hsa-miR-21-5p	Lipid transport
AKT2	hsa-miR-423-3p	Lipid transport
AKT2	hsa-miR-708-5p	Lipid transport
AKT2	hsa-miR-744-5p	Lipid transport
AKT2	hsa-miR-877-3p	Lipid transport
FABP7	hsa-miR-132-3p	Lipid transport
LDLR	hsa-let-7b-5p	Lipid transport
LDLR	hsa-miR-124-3p	Lipid transport
LDLR	hsa-miR-128-3p	Lipid transport
LDLR	hsa-miR-130a-3p	Lipid transport
LDLR	hsa-miR-130b-3p	Lipid transport
LDLR	hsa-miR-149-5p	Lipid transport
LDLR	hsa-miR-19b-3p	Lipid transport
LDLR	hsa-miR-26b-3p	Lipid transport
LDLR	hsa-miR-30a-5p	Lipid transport
LDLR	hsa-miR-335-5p	Lipid transport
LDLR	hsa-miR-744-5p	Lipid transport
LDLR	hsa-miR-92a-3p	Lipid transport
LDLR	hsa-miR-92b-3p	Lipid transport
LDLR	hsa-miR-93-5p	Lipid transport
NR1H3	hsa-miR-613	Lipid transport
NR1I2	hsa-let-7a-5p	Lipid transport
NR1I2	hsa-miR-148a-3p	Lipid transport
NR1I2	hsa-miR-16-5p	Lipid transport
NR1I2	hsa-miR-615-3p	Lipid transport
NR5A2	hsa-miR-1	Lipid transport
ARFGAP1	hsa-miR-30c-5p	LD proteins
ARFGAP1	hsa-miR-423-3p	LD proteins
ARFGAP1	hsa-miR-93-3p	LD proteins
ARG1	hsa-miR-1	LD proteins
ARG1	hsa-miR-30a-5p	LD proteins
ARG1	hsa-miR-7-5p	LD proteins
ATG7	hsa-miR-320c	LD proteins
ATG7	hsa-miR-375	LD proteins
CAV1	hsa-miR-103a-3p	LD proteins
CAV1	hsa-miR-124-3p	LD proteins
CAV1	hsa-miR-199a-5p	LD proteins
CAV1	hsa-miR-26b-5p	LD proteins
CAV1	hsa-miR-34b-5p	LD proteins
CAV1	hsa-miR-34c-5p	LD proteins
CAV1	hsa-miR-7-5p	LD proteins
CAV2	hsa-miR-199a-3p	LD proteins
CAV2	hsa-miR-26b-5p	LD proteins
CAV2	hsa-miR-93-5p	LD proteins
DYNC1H1	hsa-let-7b-5p	LD proteins
DYNC1H1	hsa-miR-122-5p	LD proteins
DYNC1H1	hsa-miR-149-5p	LD proteins
DYNC1H1	hsa-miR-221-3p	LD proteins
DYNC1H1	hsa-miR-455-3p	LD proteins
DYNC1H1	hsa-miR-615-3p	LD proteins
DYNC1H1	hsa-miR-92a-3p	LD proteins
DYNC1H1	hsa-miR-96-5p	LD proteins
DYNC2H1	hsa-let-7a-5p	LD proteins
DYNC2H1	hsa-miR-124-3p	LD proteins
DYNC2H1	hsa-miR-125b-5p	LD proteins

DYNC2H1	hsa-miR-155-5p	LD proteins
DYNC2H1	hsa-miR-331-3p	LD proteins
ERLIN1	hsa-miR-10a-5p	LD proteins
ERLIN1	hsa-miR-10b-5p	LD proteins
ERLIN1	hsa-miR-17-5p	LD proteins
ERLIN1	hsa-miR-30a-5p	LD proteins
ERLIN1	hsa-miR-30c-5p	LD proteins
ERLIN1	hsa-miR-34a-5p	LD proteins
ERLIN1	hsa-miR-615-3p	LD proteins
FITM2	hsa-miR-373-3p	LD proteins
FLOT1	hsa-miR-182-5p	LD proteins
FLOT1	hsa-miR-214-3p	LD proteins
FLOT1	hsa-miR-320a	LD proteins
FLOT1	hsa-miR-484	LD proteins
MGLL	hsa-miR-30c-5p	LD proteins
MGLL	hsa-miR-7-5p	LD proteins
NSF	hsa-miR-100-5p	LD proteins
NSF	hsa-miR-103a-3p	LD proteins
NSF	hsa-miR-16-5p	LD proteins
NSF	hsa-miR-192-5p	LD proteins
NSF	hsa-miR-193b-3p	LD proteins
NSF	hsa-miR-215-5p	LD proteins
NSF	hsa-miR-26b-5p	LD proteins
NSF	hsa-miR-877-3p	LD proteins
PNPLA3	hsa-miR-335-5p	LD proteins
RAB32	hsa-miR-124-3p	LD proteins
RAB32	hsa-miR-26b-5p	LD proteins
SNAP23	hsa-let-7b-5p	LD proteins
SNAP23	hsa-miR-124-3p	LD proteins
SNAP23	hsa-miR-34a-5p	LD proteins
SNAP23	hsa-miR-98-5p	LD proteins
STX5	hsa-miR-155-5p	LD proteins
VIM	hsa-miR-124-3p	LD proteins
VIM	hsa-miR-1301-3p	LD proteins
VIM	hsa-miR-138-5p	LD proteins
VIM	hsa-miR-16-5p	LD proteins
VIM	hsa-miR-17-3p	LD proteins
VIM	hsa-miR-26b-5p	LD proteins
VIM	hsa-miR-30a-5p	LD proteins
VIM	hsa-miR-30c-5p	LD proteins
VIM	hsa-miR-3605-3p	LD proteins
VIM	hsa-miR-615-3p	LD proteins
VIM	hsa-miR-9-5p	LD proteins
ANGPTL4	hsa-miR-1	Glucose metabolism
ANGPTL4	hsa-miR-124-3p	Glucose metabolism
CEBPA	hsa-miR-1	Glucose metabolism
CEBPA	hsa-miR-1226-3p	Glucose metabolism
CEBPA	hsa-miR-124-3p	Glucose metabolism
CEBPA	hsa-miR-125b-5p	Glucose metabolism
CEBPA	hsa-miR-138-5p	Glucose metabolism
CEBPA	hsa-miR-193a-5p	Glucose metabolism
CEBPA	hsa-miR-744-5p	Glucose metabolism
FOXO1	hsa-miR-106b-3p	Glucose metabolism
FOXO1	hsa-miR-132-3p	Glucose metabolism
FOXO1	hsa-miR-153-3p	Glucose metabolism
FOXO1	hsa-miR-15a-5p	Glucose metabolism
FOXO1	hsa-miR-182-5p	Glucose metabolism
FOXO1	hsa-miR-183-5p	Glucose metabolism
FOXO1	hsa-miR-186-5p	Glucose metabolism
FOXO1	hsa-miR-196a-5p	Glucose metabolism
FOXO1	hsa-miR-223-3p	Glucose metabolism
FOXO1	hsa-miR-27a-3p	Glucose metabolism
FOXO1	hsa-miR-335-5p	Glucose metabolism
FOXO1	hsa-miR-9-5p	Glucose metabolism
FOXO1	hsa-miR-96-5p	Glucose metabolism
FOXO1	hsa-miR-98-5p	Glucose metabolism
GK	hsa-miR-10b-5p	Glucose metabolism
GK	hsa-miR-132-3p	Glucose metabolism
HNF4A	hsa-miR-197-3p	Glucose metabolism
HNF4A	hsa-miR-24-3p	Glucose metabolism
HNF4A	hsa-miR-34a-5p	Glucose metabolism
HNF4A	hsa-miR-34b-5p	Glucose metabolism
HNF4A	hsa-miR-34c-5p	Glucose metabolism

HNF4A	hsa-miR-449a	Glucose metabolism
HNF4A	hsa-miR-629-5p	Glucose metabolism
HNF4A	hsa-miR-765	Glucose metabolism
HNF4A	hsa-miR-766-3p	Glucose metabolism
IRS1	hsa-miR-126-3p	Glucose metabolism
IRS1	hsa-miR-145-5p	Glucose metabolism
IRS1	hsa-miR-148a-3p	Glucose metabolism
IRS1	hsa-miR-152-3p	Glucose metabolism
IRS1	hsa-miR-7-5p	Glucose metabolism
IRS2	hsa-let-7b-5p	Glucose metabolism
IRS2	hsa-let-7e-5p	Glucose metabolism
IRS2	hsa-miR-145-5p	Glucose metabolism
IRS2	hsa-miR-33a-5p	Glucose metabolism
IRS2	hsa-miR-374b-5p	Glucose metabolism
IRS2	hsa-miR-484	Glucose metabolism
IRS2	hsa-miR-7-5p	Glucose metabolism
IRS2	hsa-miR-98-5p	Glucose metabolism
ACSL1	hsa-let-7b-5p	Fatty acid oxidation
ACSL1	hsa-miR-191-5p	Fatty acid oxidation
ACSL1	hsa-miR-192-5p	Fatty acid oxidation
ACSL1	hsa-miR-335-5p	Fatty acid oxidation
ACSL1	hsa-miR-34a-5p	Fatty acid oxidation
ACSL1	hsa-miR-93-3p	Fatty acid oxidation
ACSL3	hsa-miR-100-5p	Fatty acid oxidation
ACSL3	hsa-miR-221-3p	Fatty acid oxidation
ACSL3	hsa-miR-335-5p	Fatty acid oxidation
ACSL3	hsa-miR-340-5p	Fatty acid oxidation
ACSL3	hsa-miR-375	Fatty acid oxidation
CPT2	hsa-miR-34a-5p	Fatty acid oxidation
CPT2	hsa-miR-378a-3p	Fatty acid oxidation
DERL1	hsa-miR-19b-3p	Fatty acid oxidation
DERL1	hsa-miR-21-5p	Fatty acid oxidation
DERL1	hsa-miR-26b-5p	Fatty acid oxidation
DERL1	hsa-miR-30a-5p	Fatty acid oxidation
FOXA2	hsa-miR-335-5p	Fatty acid oxidation
PPARA	hsa-miR-10b-5p	Fatty acid oxidation
PPARA	hsa-miR-124-3p	Fatty acid oxidation
PPARA	hsa-miR-141-3p	Fatty acid oxidation
PPARA	hsa-miR-21-5p	Fatty acid oxidation
PPARA	hsa-miR-22-3p	Fatty acid oxidation
PPARA	hsa-miR-335-5p	Fatty acid oxidation
PPARA	hsa-miR-519d-3p	Fatty acid oxidation
GLUD1	hsa-miR-185-5p	Bio-markers
GLUD1	hsa-miR-193b-3p	Bio-markers
GLUD1	hsa-miR-93-3p	Bio-markers
GLUD1	hsa-miR-99a-5p	Bio-markers
GOT1	hsa-miR-769-5p	Bio-markers
